# Electrochemical Intercalation Reaction of Sodium into the Layered Transition Metal Dichalcogenide ZrS_2_—Influence of the Electrolyte Solvent

**DOI:** 10.1021/acs.inorgchem.5c02331

**Published:** 2025-08-27

**Authors:** Lina Liers, Liuda Mereacre, Hang Li, Julia Mickenbecker, Michael Knapp, Sylvio Indris, Malte Behrens, Sebastian Mangelsen

**Affiliations:** † Institute of Inorganic Chemistry, 9179 Kiel University, Max-Eyth-Str. 2, Kiel 24118, Germany; ‡ Institute for Applied Materials - Energy Storage Systems, 28335 Karlsruhe Institute of Technology, P.O. Box 3640, Karlsruhe 76021, Germany; § Applied Chemistry and Engineering Research Centre of Excellence (ACER CoE), Université Mohammed VI Polytechnique (UM6P), Lot 660, Hay Moulay Rachid, Ben Guerir 43150, Morocco

## Abstract

While TiS_2_ has been extensively studied for its ability to intercalate alkali metals like Li or Na, the higher homologue ZrS_2_ was studied only sparsely. Furthermore, an influence of different coordinating and noncoordinating electrolyte solvents on cyclability as well as the structural changes of the host structures had been observed for different active materials. In this study, we therefore investigated the intercalation mechanism of Na^+^ ions into layered 1T-ZrS_2_ using electrolytes with solvents of different coordination strengths toward Na^+^, namely sodium trifluoromethanesulfonimide in ethylene carbonate and diethyl carbonate (1:1, EC/DEC) and sodium triflate in bis­(2-methoxyethyl) ether (diglyme). At low intercalation degrees, coordinating solvents (e.g., diglyme) lead to a cointercalation in combination with a large expansion of the interlayer distance. After deintercalation, a turbostratically disordered material was obtained. In contrast, for weakly coordinating solvents (EC/DEC) no cointercalation was observed which enabled us to observe the reversible phase transitions from 1T-ZrS_2_ to 3R-NaZrS_2_ upon (de)­intercalation. This transition proceeds via stacking faults and was analyzed in detail. Further, the intermediates of the electrochemical intercalation were analyzed by solid-state NMR and cycling stability tests were carried out. The long-term stability of cells prepared from ZrS_2_ is comparable, independent of the electrolyte solvent.

## Graphical abstract

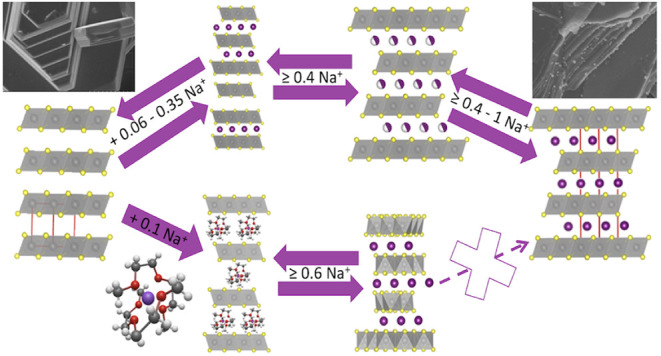

## Introduction

Electrochemical intercalation of alkali metal ions has been of general interest since the beginning of mass production of lithium-ion-batteries (LIBs). At a time when the effects of climate change are becoming increasingly noticeable, the pressure to research alternative energy sources and electrified transport is increasing.^1^ High energy densities, long cycle life, high output voltages, and an overall energy efficiency of up to 70% are only few advantages of rechargeable batteries.^2–5^ Besides commercially used electrodes of transition metal oxides (TMOs), alternative materials such as transition metal dichalchogenides (TMDCs) have shown intriguing and promising properties.^6–8^ Among those TMDCs, TiS_2_ was the first electrode material tested in LIBs and was intensively investigated because of promising cycle stability and a high specific capacity.^9^ Its benefits are the low cost and high abundance as well as the outperforming cycle stability and a theoretical specific capacity of 239 mA h g^–1^.^10^ Further, sodium intercalation in TiS_2_ is also possible which makes this material interesting for sodium-ion-batteries (SIBs).^11–15^ In contrast to their Li analogues (one-step voltage profile) the electrochemical intercalation of Na^+^ results in a multistep voltage profile which indicates multiple phase transitions of Na_*x*_TiS_2_. Thus, the capacity of sodium intercalated TiS_2_ fades faster than for the Li analogues.^16,17^

While flat plateaus indicate a region where two different phases coexist, steps indicate a stronger increase in the chemical potential at the end of a reaction that represents a phase transition or different cation ordering schemes within the layers.^16^ Repeated phase transitions and thus rearrangements of the structural units result in stress on the material leading to an incomplete deintercalation of sodium ions causing capacity loss and low cyclability. A detailed study of the sequential structural changes is given in refs 12,18,19 . Furthermore, in several studies a strong dependency of the cycling stability as well as the crystallinity on the electrolyte solvent was observed. Multicoordinating and sterically demanding solvents such as bis­(2-methoxyethyl)­ether (diglyme) cointercalate into the layered structure resulting in a strong expansion of the interlayer spacing.^20–24^ This phenomenon can be prevented using saturated solutions of the conducting salt in an appropriate solvent or the cointercalation is used to build a cointercalation battery cell with e.g., graphite as anode and NaTiS_2_ as cathode.^20,25,26^

As some structural chemistry is involved in the study, a brief introduction in the relevant phases 1T-ZrS_2_ and 3R-NaZrS_2_ is given at this stage. The combination of number and letter, e.g., 1T, is known as Ramsdell notation^27^ and indicates the number of layers in a unit cell and its symmetry with T: trigonal, H: hexagonal and R: rhombohedral. ZrS_2_ crystallizes in the 1T polytype (*P*3̅*m*1) which is referred to as CdI_2_ structure type, where Zr occupies the octahedral sites of hexagonally closed packed sulfur anions. This results in a primitive stacking sequence of AbC­[]_T_AbC··· where capital letters describe sulfur atom sites and lower letters describe metal atom sites, the empty brackets indicate a tetrahedral vacancy in the gap (see Figure 1 for the crystal structures and stacking patterns).^12,19,28^ The ZrS_6_ octahedra are edge-sharing and the layers of anions are held together by van der Waals forces. When sodium is intercalated, the resulting polytype of NaZrS_2_ (*R*3̅*m*, α-NaFeO_2_ type) is the 3R_a_ (there are multiple variants of the 3R type, cf. the article on TMDCs by Katzke et al.^29^) in which the layers are shifted in the *a*/*b* plane compared to 1T-ZrS_2_. The Zr ions still occupy the octahedral sites but in a cubic closed packing of sulfur atoms. The resulting stacking sequence is AcB­[Na]_O_CbA­[Na]_O_BaC··· Sodium ions are intercalated into octahedral sites in the van der Waals gaps.^19,28^ Note that the intercalation of sodium brings about a marked expansion of the interlayer space (2.91 to 3.74 Å, cf. Figure 1), while the thickness of the ZrS_2_ slab increases only slightly (2.91 to 3.05 Å).

**Figure 1:**
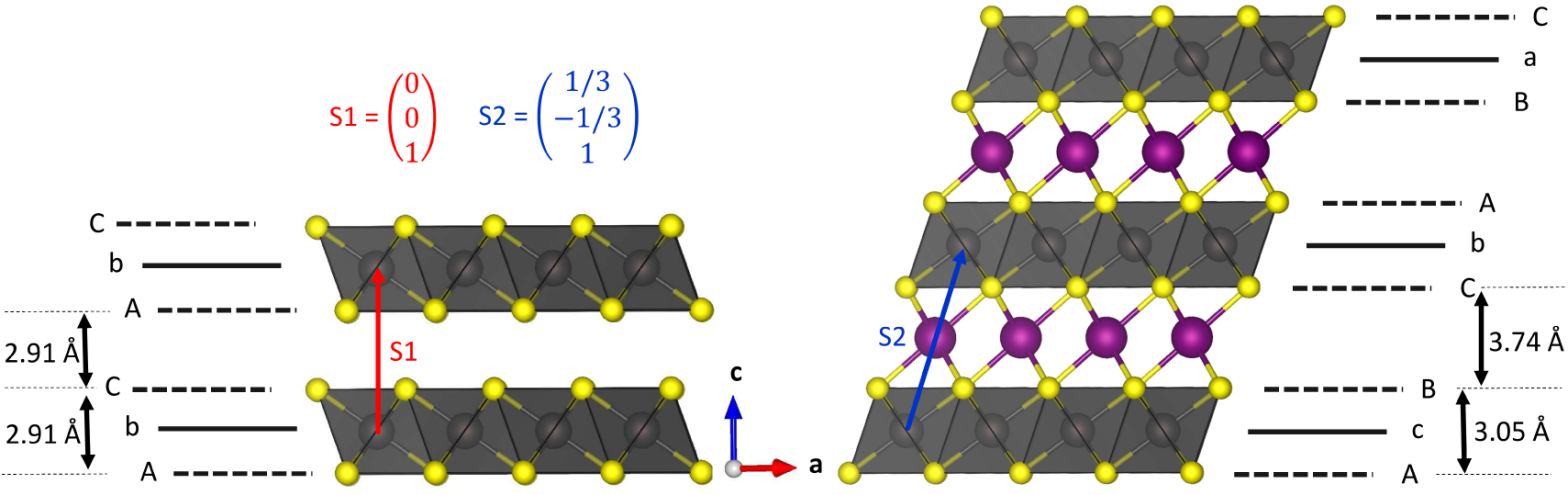
Scheme showing the stacking in the crystal structure of 1T-ZrS_2_ (left) and 3R-NaZrS_2_ (right), the stacking vectors S1 and S2 (components in *a*, *b*, and *c* direction) as well as stacking order of the anions and cations. Further the interlayer space and thickness of the ZrS_2_ (as derived from the z-component of the sulfur atoms in the crystal structures) are indicated.

Higher homologues and isostructural compounds of TiS_2_ such as ZrS_2_ are underexplored in terms of electrochemical sodium intercalation and the underlying reaction mechanism. For the example of ZrS_2_ only a few studies were dealing with electrochemical intercalation of lithium. In these reports, a reversible phase transition from the 1T-ZrS_2_ structure to the 3R-LiZrS_2_ structure was observed during the intercalation of lithium ions.^25,28,30^ This is in line with the results of chemical lithium intercalation using a solution of lithium in ammonia^12^ or *n*-butyllithium.^31–33^ For the intercalation of sodium ions into ZrS_2_ as host structure only chemical intercalation experiments were performed using a solution of sodium in ammonia. A phase transition from 1T-ZrS_2_ to 3R-NaZrS_2_ was also observed during the intercalation of sodium.^12,34,35^ These observations can be explained by the structure of the materials. Generally, when intercalating Na^+^ instead of Li^+^ more phase transitions or ordering processes are observed, which is likely linked to the significantly larger ionic radius of the sodium ions. Further, studies revealed an influence of the electrolytes on structural changes during electrochemical intercalation of lithium into ZrS_2_. While 1 M electrolyte solutions lead to a cointercalation of strong coordinating solvent molecules into the TMDC layers, for weakly coordinating electrolyte solvents no indication of cointercalation could be observed.^20,36,37^ Saturated solutions are assumed to prevent solvent cointercalation.^25^ However, structural investigations on intermediates formed during the electrochemical intercalation of sodium ions and cointercalation of solvent molecules into the host structure ZrS_2_ are underexplored. Since the ability of ZrS_2_ to intercalate sodium ions is known, but the underlying reaction mechanism and the influence of different electrolyte solvents are not, the following report presents the results of the investigation of phase transformations during the electrochemical reaction as a function of the electrolyte solvent. To figure out the electrochemical (de)­intercalation reaction pathway we performed complementary *in situ*- and *ex situ* XRPD measurements as well as magic-angle spinning (MAS) NMR and cycling stability experiments.

## Experimental Section

### Synthesis

ZrS_2_ was synthesized by high temperature synthesis. Zirconium (H. C. Starck Tungsten Powders) and sulfur (Chempur, 99.999%) were mixed in stoichiometric amounts and sealed in a quartz glass ampule (*p* < 10^4^ mbar). The ampule was placed in a muffle furnace, heated to 723 K within 1 d, and maintained at this temperature for 1 d. Afterward, the temperature was increased to the reaction temperature of 1073 K within 1 d, maintained for 3 d and cooled to room temperature naturally.

### Electrochemical Intercalation

All preparation steps were performed under inert conditions in an argon filled glovebox (99.999% Ar, MBraun Unilab, <1 ppm of H_2_O, <1 ppm of O_2_). Electrochemical cells (powder cells) with material designated for *ex situ* XRPD were assembled as follows: ZrS_2_ was mixed with conducting carbon (SUPER C65, Timcal, Switzerland) in a ratio of 70:30 wt %. Around 30 mg of the mixture was pressed into a pellet with a diameter of 8 mm and placed in Swagelok type test cells. The pellet was covered with a separator (Separion S240P30, Litarion) and two glass fiber filters (Whatman, United Kingdom). Pure sodium metal was used as counter electrode. To study the effect of electrolyte solvents, 1 M NaCF_3_SO_3_ (NaOTf, abcr, 98%) in bis­(2methoxyethyl)­ether (Diglyme, Acros Organics, 99+%, extra dry) and 1 M NaC_2_HF_6_NO_4_S_2_ (NaTFSI) in ethylene carbonate and diethyl carbonate (EC:DEC; 1:1) (solvionic) were used as electrolytes, respectively.

For electrochemical cyclization, electrodes were prepared by mixing ZrS_2_ with SUPER C65, polyvinylidenefluoride (PVDF 5301/1001, Solvay) in a ratio of 80:10:10 wt % and suspended in *N*-methyl-2-pyrrolidone (NMP, Fischer Reagents, 99.8%). The suspension was mixed in a MM400 ball mill (Retsch) for 10 min at 10 Hz and cast onto Cu foil using the doctor-blade method (*h* = 150 μm). The electrodes were dried at room temperature for 4 h and finally in a vacuum oven at 323 K overnight. Afterward, circular electrodes with a diameter of 10 mm were punched out and inserted into Swagelok type test cells. The cell assembling was carried out as described above. Additionally, sodium hexafluorophosphate (NaPF_6_) in propylene carbonate (PC) and NaOTf in Tetrahydrofuran (THF) were used as electrolytes.

A Neware 8 channel battery analyzer was used for galvanostatic cyclization within a voltage range of 2.8 to 0.3 V and 2.8 to 1.0 V respectively, applying a current rate of C/20 for powder cells and C/5 for film cells. The electrochemical reaction was interrupted at different sodium uptake/release steps to investigate the intercalation reaction mechanism. Afterward, the electrodes were recovered, washed with diglyme or *n*-Hexane depending on the electrolyte used and dried in an argon-filled glovebox. The dry powder was filled in borosilicate capillaries (Hilgenberg, Type No. 10, Ø = 0.5 mm) and sealed under argon atmosphere. The film electrodes were washed in the same way as the powder electrodes. For the XRPD measurements, the material was transferred from the copper foil to a Kapton foil.

For the *in situ* XRPD measurement, a custom-made coin cells with a center hole for transmission of the X-ray beam was used (Figure S1).^38^ The upper and lower housings holes were sealed with Kapton foil (*h* = 50 μm). The working electrode was placed on a carbon cloth and a sodium–carbon composite was used as the counter electrode. The electrodes were separated by a Whatmann borosilicate separator which was impregnated with 200 μL of 1 M NaTFSI in EC:DEC (1:1) electrolyte. The cell was sealed in a cell press with a pressure of 650 psi.

### Material Characterizations

Powder X-ray diffraction (XRPD) of *ex situ* powder cells and film electrodes of the cycling experiments were performed in transmission geometry using a PANalytical Empyrean (Cu K_α_ radiation (λ = 1.54184 Å), focusing X-ray mirror, PIXcel 1-D detector).

*In situ* XRPD of electrochemical cells was performed using a dedicated *in-operando* setup.^39^ The diffractometer (STOE STADI P) has a Ag source (λ = 0.55942 Å) with step width 0.015° 2θ, Ge111 curved monochromator and a DECTRIS Mythen 2 2K detector. During the measurement, there was a brief interruption of 60 min after 39.5 h of measurement time.

^23^Na magic-angle spinning (MAS) nuclear magnetic resonance (NMR) spectroscopy was performed with a Bruker Avance neo 200 MHz spectrometer at a magnetic field of 4.7 T, corresponding to a Larmor frequency of 52.9 MHz. Spinning was performed in 1.3 mm rotors at 55 kHz. Spectra were acquired with a rotor-synchronized Hahn-echo pulse sequence with a π/2 pulse length of 0.92 μs and a recycle delay of 1 s. Spectral intensities were normalized with respect to the sample mass and the number of scans. The ^23^Na NMR shifts are referenced to an aqueous 1 M NaCl solution at 0 ppm.

All structure refinements were carried out using TOPAS Academic V6.0.^40^ Instrumental line broadening was described using the fundamental parameters approach as implemented in TOPAS^41^ and cross-checked against a measurement of a line shape standard material (LaB_6_ SRM 660c). The crystal structure of Na_*x*_ZrS_2_ was obtained by Rietveld refinement^42^ using the isotypic crystal structure of NaTiS_2_ ^43^ as starting model. Both samples from electrochemical intercalation and high temperature synthesis with slightly different occupancies of sodium were used. The CIF files are deposited as CCDC 2470929 and 2470930, some crystallographic and refinement data can be found in Table S1.

Scanning electron microscopy (SEM) in combination with energy-dispersive X-ray spectroscopy (EDX) was performed on a Hitachi SU8700 equipped with an Oxford EDX detector Ultim 100. The samples were prepared on carbon stripes on an aluminum pin mount sample holder in an argon filled glovebox and transferred to the microscope. Images were recorded using three different detectors, e.g., an in-column secondary electron (SE) upper detector (UD), an in-column backscattered electron (BSE) middle detector (MD) and an Everhart-Thornley detector in the chamber (SE/BSE, lower detector LD).

## Results and Discussion

### Characterization of Pristine ZrS_2_

The product obtained from solid state reaction from the elements was analyzed via SEM and XRPD measurements. SEM images showed hexagonally shaped crystals with a plate-like, layered morphology (Figures S2 and 7a). The comparison of the observed XRPD pattern and data from the literature confirms the formation of 1T-ZrS_2_,^44^ which crystallizes in the trigonal space group *P*3̅*m*1 (Figure 2). Nevertheless, a secondary phase of ZrOS can be observed in the XRPD pattern (Figure S3). The origin of ZrOS is a side reaction of zirconium with the quartz glass ampule or traces of water released from the quartz. From a Rietveld refinement, the latter was quantified to amount to 0.3 wt % of the pristine sample. The lattice parameters of 1T-ZrS_2_ *a* = 3.6596(2) Å, *c* = 5.8243(5) Å and the cell volume of 67.55(4) Å^3^ are consistent with the literature.^45–49^

**Figure 2:**
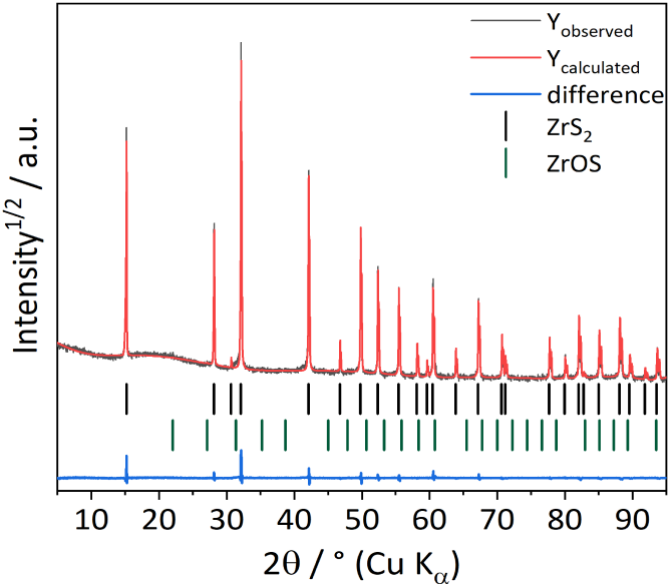
Difference plot of the Rietveld refinement for the sample obtained by high temperature synthesis.^44^ *r*_wp_ = 9.71%, gof = 1.21, ZrOS 0.3 wt %.

After the preparation of the sample for electrochemical measurements e.g., mixing ZrS_2_ and conducting carbon C65 in a ball mill, the quantified amount of ZrOS raises up to 2.2 wt %. The XRPD pattern (Figure S3) shows a broadening of the reflections assigned to ZrS_2_ as a result of the mechanical stress and the intensity of the reflections attributable to ZrOS is increased. The increased weight percentage of ZrOS is likely not introduced by a reaction during the ball milling (carried out under inert conditions), rather it may be argued that the ball milling affects the overall crystallinity of the ZrS_2_ and thus increases the weight percentage of ZrOS in the crystalline part of the sample.

### Intercalation Mechanism and the Influence of Electrolyte Solvents

Different electrolytes were studied in order to elucidate their influence on the intercalation mechanism of sodium in ZrS_2_. First, we briefly highlight observed differences based on the potential curves of the first discharge. Thereafter we will discuss the results of *ex situ* experiments performed at specific points in those profiles. When comparing the first discharge using the electrolytes 1 M NaTFSI in EC:DEC and 1 M NaOTf in diglyme, a two-step profile is observed when using the diglyme based electrolyte, in contrast to a one-step profile by using the EC:DEC based electrolyte (Figure 3a). The cyclic voltammetry (CV) profile of the cell cycled with EC:DEC as electrolyte solvent shows an intensive reduction peak at 1.5 V which fits the intercalation plateau of the galvanostatic voltage profile. Further, a broad reduction peak at 1.2 V and another reduction peak at 0.5 V belonging to the changes in slope of the voltage profiles are visible. The corresponding oxidation peaks at 1.4, 1.6 and 1.8 V indicate the reversibility of the redox reactions (Figure 3b). Further, cells of ZrS_2_ vs Na^+^|Na using the diglyme based electrolyte can be discharged to a potential of 0.1 V which corresponds to an uptake of 1.2 Na^+^/fu. while for cells of ZrS_2_ vs Na^+^|Na using the carbonate based electrolyte only an uptake of 1 Na^+^/fu. can be obtained (Figure 3a).

**Figure 3:**
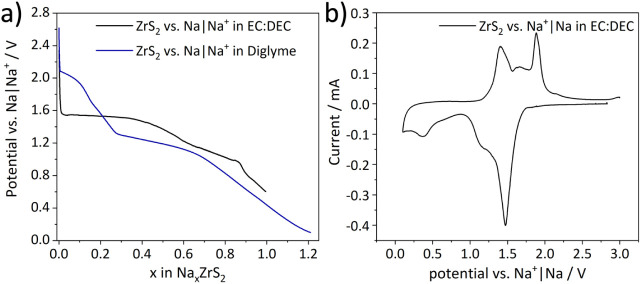
(a) Comparison of first discharge curves for Na^+^ intercalated in ZrS_2_ using 1 M NaTFSI in EC:DEC (black) and 1 M NaOTf in diglyme (blue) as electrolyte, respectively. (b) Corresponding CV curve of the cell cycled using 1 M NaTFSI in EC:DEC.

The initial discharge curve of ZrS_2_ vs Na^+^|Na using diglyme as electrolyte solvent shows one short pseudoplateau at 2.2 V vs Na^+^|Na until an uptake of 0.1 Na^+^/fu. and an extended one at 1.25 V vs Na^+^|Na from an uptake of 0.3–0.7 Na^+^/fu. (Figure 4a). The plateaus indicate two phase regions while regions with a gradient indicate presence of a single-phase region. For this specific example it means that a mixture of two different phases coexists at a potential of 2.2 V followed by a phase transition and a single-phase region when the potential drops to 1.25 V. At this potential a second pseudo plateau can be observed which indicates another two-phase region. When the potential drops after an uptake of 0.7 Na^+^/fu. the final 3R-Na_*x*_ZrS_2_ phase is filled up with Na^+^ until a maximum uptake of 1.2 Na^+^/fu. This may indicate side reactions like decomposition of the electrolyte or the onset of a conversion type reaction as the maximum amount of intercalated sodium in the ZrS_2_ structure is 1 Na per fu. based on the available octahedral vacancies in the interlayer space. Since it was possible to discharge the electrochemical cell to a low potential of 0.3 V, side reactions like the decomposition of the electrolyte and a following formation of a solid electrolyte interphase (SEI) are possible.

**Figure 4:**
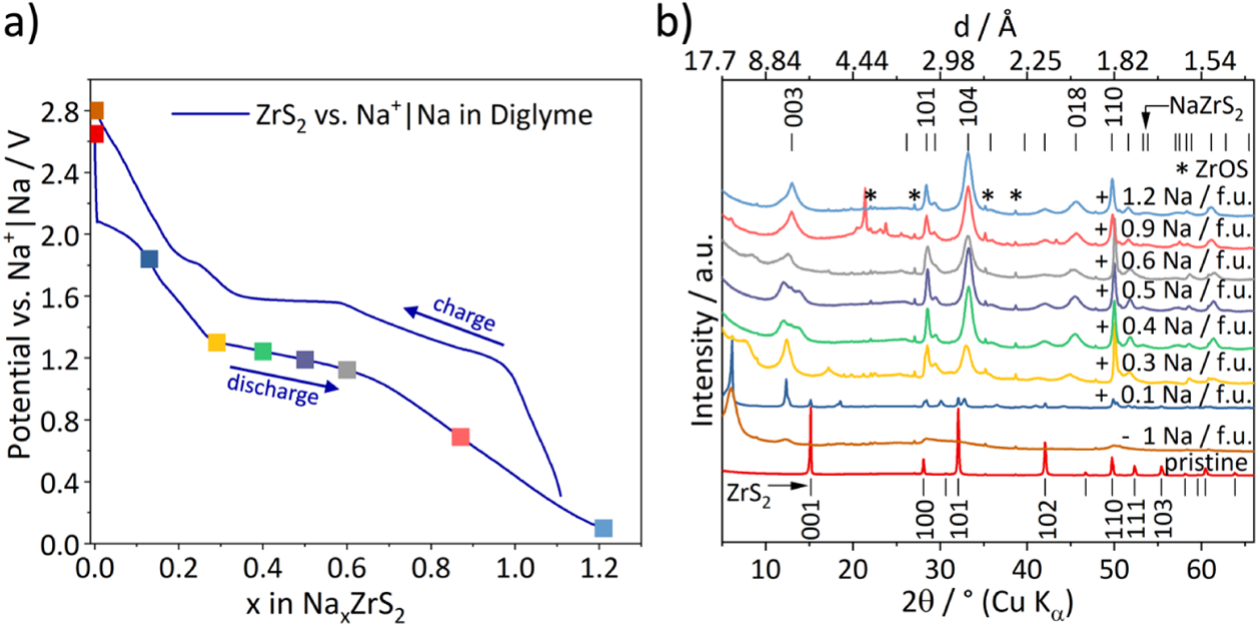
(a) Voltage profile of the first cycle using a diglyme-based electrolyte where colored squares mark the points where *ex situ* XRPD pattern were collected, which are shown in (b). ZrOS impurities originating from the solid state synthesis are marked with asterisks.

We will now briefly comment on the possible redox events upon intercalation. For the related system Li_*x*_TiS_2_ there is evidence for charge transfer from Li to both Ti and S,^50^ further only a partial charge transfer from Li to the host appears to take place.^51^ Knowing that for Zr compared to Ti the oxidation state +IV is even more stable, it seems likely that the intercalation of Na in ZrS_2_ leads to a similar situation of charge transfer to both elements, that goes beyond the assignment of formal oxidation states. Referring to Figure 1 the thickness of the layer (distance between top and bottom rows of sulfur atoms) increases only slightly upon intercalation of sodium, which translates to an increase of the Zr–S bond length from 2.568 Å in our pristine material to 2.608 Å in the electrochemically intercalated Na_0.95_ZrS_2_, which is only a slight increase. The discussion of the oxidation states remains open and may motivate further studies.

We will now discuss the XRPD measurements performed at different states of Na uptake. These show (Figure 4b) that for cells cycled with diglyme additional very intense and sharp Bragg reflections at 6.1° (*d* = 14.4 Å), 12.35° (*d* = 7.2 Å) and 18.6° 2θ (*d* = 4.8 Å) can be observed for a sodium uptake of 0.1 Na^+^/fu., which corresponds to the end of the first pseudo plateau. These are likely 00*l* reflections, corresponding to a large expansion of the interlayer spacing. The *d*-spacings match according to 00*l*, 00*l* + *1* and 00*l* + *2*, indicating they belong to one phase. The pronounced expansion of the structure along the *c*-axis for low degrees of intercalation can be understood by a cointercalation of diglyme molecules with the sodium ions (likely as sodium–diglyme complex) as it was published for a recent study on Na^+^ intercalation in TiS_2_ using a diglyme based electrolyte.^20^ It should be noted that this sample still contains some pristine ZrS_2_, which is likely some yet unreacted material and completion of the reaction for all material may require a slightly lower cutoff potential. To check whether the cointercalation is a kinetic effect, we measured the same sample again after three years and found that the reflection positions are still the same, i.e., the cointercalated phase was stable (Figure S4).

For an uptake of 0.3 Na^+^/fu. some of the additional reflections shift to smaller *d*-spacings and the reflections broaden anisotropically. A vast majority of the reflections can be assigned to Na_*x*_ZrS_2_, with cross plane reflections being broadest (e.g., 104, 33.2° 2θ) and sharp reflections corresponding to lattice planes in plane (e.g., 110, 49.8° 2θ). This is indicative of stacking disorder, while the overall broadening may also correspond to a smaller crystallite size or strain effects. In addition, a series of low-intensity reflections occur, which can be assigned to a ZrOS byphase (22.0°, 27.1°, 35.2°, 38.7° 2θ) that is formed during synthesis (Figure S3). The reflections between 18.5° and 22.0° 2θ originate from residues of the separator that could not be completely washed off. Two additional reflections at 7.9 and 17.3° 2θ cannot be assigned to ZrS_2_ or NaZrS_2_ and are not integrals of the 00*l* reflections. Possibly they correspond to an intermediate state. This can be proposed since the shrinking cell volume occurring after the potential drop down to 1.3 V indicates the diglyme molecules being fully or partly deintercalated to enable more space for Na^+^ intercalation. By further Na^+^ intercalation up to 0.4–0.5 Na^+^/fu. (within the extended pseudo plateau) the reflection at 12.5° 2θ (*d* = 7.1 Å; 0.3 Na^+^/fu.) now exhibits shoulders to both higher and lower *d*-spacings, indicating different interlayer spacings and thus different intercalation degrees. When going from 0.6 to 0.9 of intercalated Na^+^ per fu. the increased slope of the discharge curve indicates a cross over to intercalation in a single phase. The XRPD pattern still corresponds to the NaZrS_2_ type phase with the reflection around 12.5° 2θ becoming more symmetric and only one shoulder is visible. However, the profile is of an unusual triangular shape, which may point to an inhomogeneous distribution of *d*-spacings and thus Na content in the interlayer space. Additionally, several intensive reflections between 20 and 30° 2θ are observed which are caused by residues of the separator after the washing process. Further Na^+^ uptake up to 1.2 per fu. leads to no further significant changes in the XRPD patterns.

After the deintercalation of Na^+^ (i.e., recharge of the cell) a phase of low crystallinity can be observed. The Bragg reflections at 6.1 and 12.4° 2θ reappear and reflections with asymmetric line shapes can be observed at 27° and 49° 2θ, corresponding to the position of the 100 and 110 reflections of 1T-ZrS_2_ (Figure S5). This particular peak shape (Warren type) is indicative of significant turbostratic disorder, i.e., random shifting or rotation of layers against each other.^52^ This pattern again points to a significantly increased interlayer spacing, which is in line with not having all Na^+^ deintercalated during the charge process. Likely some diglyme is reintercalated in the interlayer spacing, causing its swelling. The (de)­intercalation process is depicted schematically in Figure 5. Starting from the 1T-ZrS_2_ phase the intercalation of Na^+^ dissolved in diglyme leads to a cointercalation of a Na–diglyme complex (+0.1–0.3 Na^+^/fu.) resulting in a large interlayer spacing. During further intercalation of Na^+^ the diglyme shell is deintercalated and 3R-Na_*x*_ZrS_2_ is formed. Due to the previous large expansion of the interlayer spacing and subsequent collapse (+0.6–1.2 Na^+^/fu.), a turbostratic disordering of the layers occurs. When sodium ions are deintercalated a cointercalated phase remains (Figure 4). The interlayer distance of the cointercalated Na_*x*_ZrS_2_ is 11.4 Å which was calculated by subtracting the thickness of ZrS_2_ in NaZrS_2_ (3.0 Å) from the d-value obtained from the XRPD measurement (14.4 Å). A cointercalation of the Na–(diglyme)_2_ complex with a size of 8.3 Å is therefore possible.

**Figure 5:**
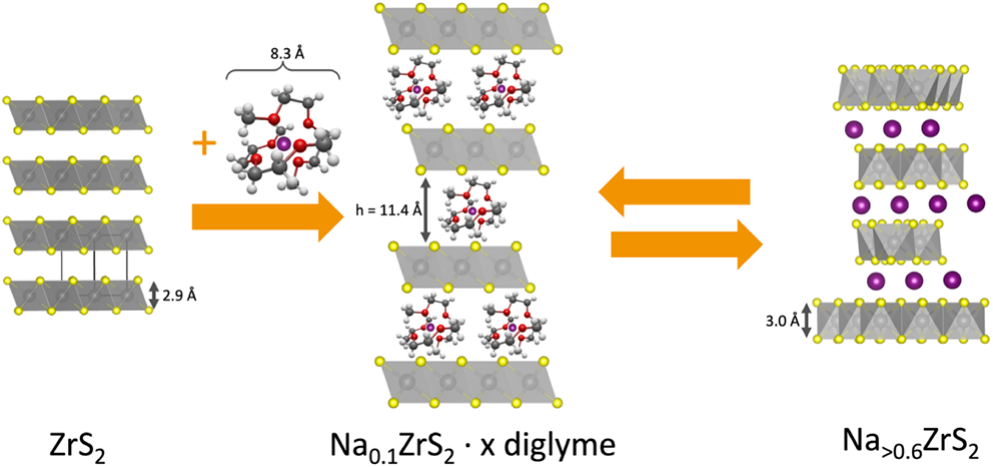
Schematic representation of a possible (co)­intercalation mechanism during the intercalation of sodium ions using a diglyme-based electrolyte.

Further samples were intercalated using the noncoordinating solvent mixture of EC:DEC and then washed with diglyme. The XRPD results (Figure S6) show that for samples intercalated with 0.06 and 0.2 Na^+^ per fu. again a pronounced expansion along the *c*-axis can be observed, indicating solvation of the intercalated cations by diglyme during washing. For higher intercalation degrees the samples do not react with diglyme. The “reactive compositions” where sodium in Na_*x*_ZrS_2_ reacts with diglyme are similar to those where the volume expansion is observed when directly cointercalating with an electrolyte containing diglyme complexes. This points to a general compositional region where the cointercalation of diglyme is thermodynamically favorable. To proof the stability of ZrS_2_ and NaZrS_2_ in the electrolyte, the synthesized powder samples were suspended in the electrolyte NaOTf in diglyme for several days. After filtration and drying, XRPD measurements were performed which give no indication of intercalation of the electrolyte solvent (Figure S4). Consequently, the cointercalation of solvent molecules in this system is triggered by the application of an electrical potential.

In contrast, the voltage profile of the initial discharge using a carbonate mixture of EC:DEC (1:1) as electrolyte solvent shows only one plateau at 1.6 V vs Na^+^|Na (Figure 6a), which extends to an uptake of ∼0.4 Na^+^ per fu. Thereafter the voltage drops rather continually down to 0.6 V, corresponding to an uptake of 1 Na^+^ per fu.

**Figure 6:**
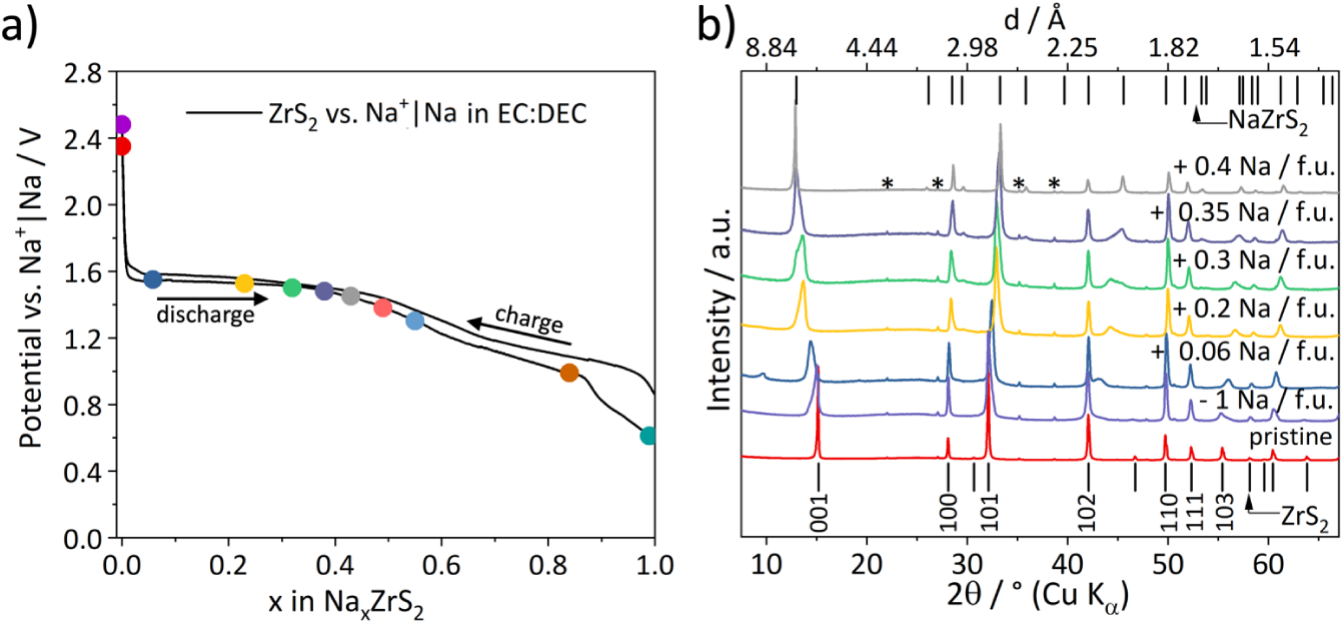
(a) Voltage profile of the first cycle using a EC:DEC-based electrolyte and (b) *ex situ* XRPD patterns recorded after a defined Na uptake that are marked with colored dots in the voltage profile. Patterns corresponding to an uptake of >0.4 Na^+^ per fu. are shown in Figure S7. Reflections of the minor impurity ZrOS are marked with an asterisk.

For ZrS_2_ cycled with EC:DEC as electrolyte solvent the crystallinity is maintained during the first discharge and even after recharge. Further, no abrupt jumps in the interlayer spacing are present, thus likely no solvent is cointercalated. Rather the intercalation mechanism is gradual as it is evident from the data in Figure 6b. For an uptake of up to 0.4 Na^+^ per fu. a transition from the 1T-ZrS_2_ type structure to 3R-Na_*x*_ZrS_2_ can be observed. Already for low Na^+^ uptake (0.06 per fu.) the XRPD pattern shows a broadening of the reflections except for *h*00 and *hk*0 reflections. The 001 reflection develops an anisotropic shape, indicating a randomized distribution of distinct interlayer spacings, furthermore a weak reflection around 10° 2θ might indicate staging but is only observed in this early stage of intercalation. Additional very broad reflections (most pronounced at 43.13° 2θ) develop which are neither in line with the structure of 1T-ZrS_2_ nor 3R-Na_*x*_ZrS_2_ and can be regarded as originating in their position from cross plane reflections of 1T-ZrS_2_. These reflections shift to higher diffraction angles upon further intercalation (up to 0.35 Na^+^ per fu.) and finally merge into cross plane reflections of 3R-Na_*x*_ZrS_2_. The 00*l* reflection at 15.16° 2θ (*d* = 5.7 Å) shifts to smaller angles during expansion of the cell along the *c*-axis by further Na^+^ intercalation. These reflections remain asymmetric, indicating multiple distinct *d*-spacings of the interlayers while transitioning between the two structure types. After intercalating 0.4 Na^+^ per fu. the reflections become sharp again, which indicates that the line broadening observed is not due to overall loss of crystallinity but rather caused by intermediate loss of long-range order. It is likely that the transition between the two structure types proceeds via stacking faults. Both structures are comprised of the same layer type, with a primitive stacking in case of 1T-ZrS_2_ and a shift of 2/3 and 1/3 along the *a* and *b* directions respectively for the 3R-type.

Upon further intercalation of Na^+^ (>0.4 Na^+^/fu., Figure S7) the 3R-Na_*x*_ZrS_2_ structure is retained and the observed small expansion of the unit cell (cf. Table 1) and change in relative intensities indicate further intercalation. The maximum amount of intercalated Na^+^ is +1 Na^+^ per fu. which is in line with the expected possible uptake of sodium in the ZrS_2_ lattice. Compared to the intercalation using a diglyme based electrolyte there are no side reactions like decomposition of the electrolyte or similar reactions.

The transition between the two structures is reversible, which is evidenced by the XRPD pattern taken after recharging of the cell. However, some line broadening, asymmetry of the 001 reflection and some shoulders (e.g., at the 102 and 103 reflections of 1T-ZrS_2_) indicate that not all Na^+^ is extracted causing some stacking faults. The phase transition of a 3R intercalated structure to a 1T deintercalated structure was also observed for 3R-Li_*x*_TiS_2_ obtained from high temperature synthesis, which was deintercalated electrochemically. The phase transition takes place at *x* < 0.4 in Li_*x*_TiS_2_ which is in line with our results for the phase transition of 1T-ZrS_2_ to 3R-Na_*x*_ZrS_2_ at an uptake of 0.4 Na^+^/fu.^53^

The SEM images show that even after the intercalation of Na^+^, intact layer packages with clear facets are retained. The individual particles are partially encased in conductive carbon (Figure 7). According to this, the (de)­intercalation of Na^+^ has no significant influence on the crystal morphology within the first discharge. EDX mapping show a uniform distribution of the elements and the embedding of Na_*x*_ZrS_2_ platelets in conductive carbon. However, finely distributed sodium can still be detected even after recharging the electrochemical cell, i.e., deintercalation of Na^+^ (Figures S8,S9). EDX measurements confirm the composition of Na_0.4_ZrS_2_ (Figure 7b) and NaZrS_2_ (Figure 7c).

**Figure 7:**
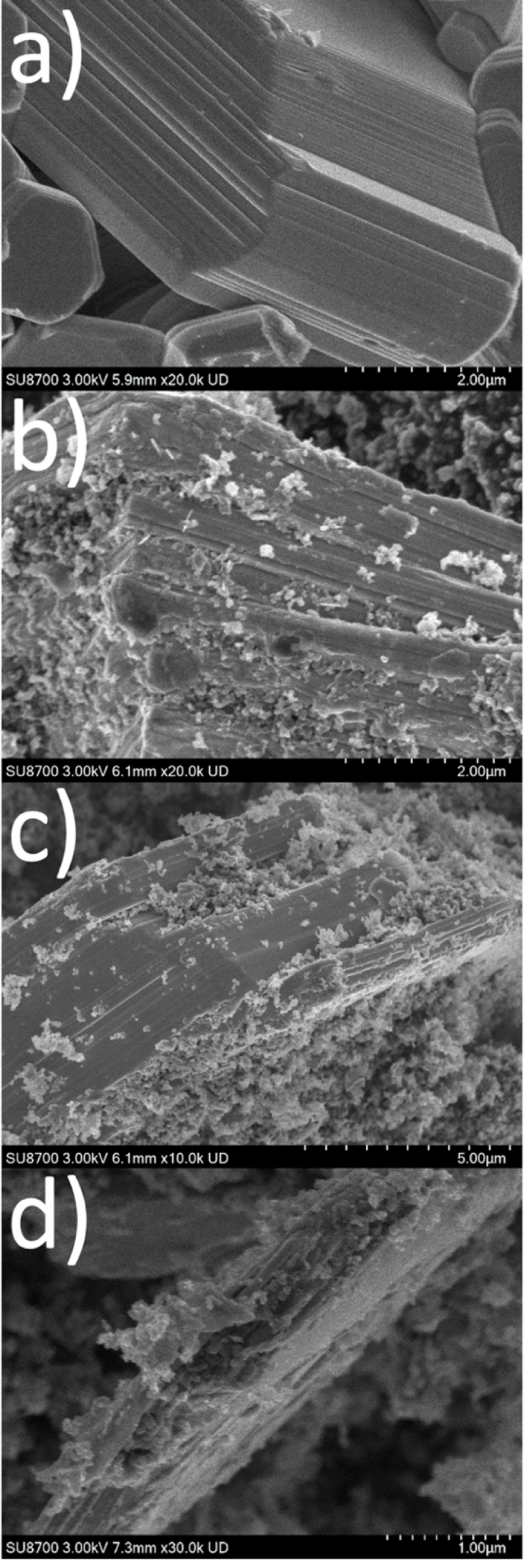
SEM images of as synthesized ZrS_2_ (a), intercalated Na_*x*_ZrS_2_ with *x* = 0.4 (b), *x* = 1 (c) and after the deintercalation of 1 Na^+^ per fu. (d).

### Detailed Analysis of the Transition from 1T-ZrS_2_To 3R-Na_*x*_ZrS_2_Via Stacking Faults

The XRPD patterns presented in Figure 6 (ZrS_2_ vs Na^+^|Na in EC:DEC) show a peculiar coexistence of sharp and very broad reflections, which are further neither fully in line with the patterns of 1T-ZrS_2_ nor 3R-Na_*x*_ZrS_2_. In order to check for stacking faults as explanation for these patterns, simulations of XRPD patterns for this structural transition were carried out using the algorithm implemented in TOPAS.^54^ An example input file is provided as Supporting Information. A continuous transition between both structures is possible by simply shifting the layers, increasing the layer distance and adding sodium in the interlayer space when 3R type stacking is present. For the simulations interlayer spacings taken from the ideal structures were kept constant. Further, *a* and *b* were fixed since these lattice parameters change only slightly during intercalation. The only variable changed was the fault probability pa, Table S2 gives the transition probabilities among the layers for this faulting scenario.

In Figure 8a the simulated patterns are shown. These illustrate that the key features observed experimentally are reproduced: Broadening of the 00*l*-reflections for frequent faulting between both structures (pa near 0.5), virtually unchanged position of the 100 reflection of 1T-ZrS_2_ (changing into the 101 in 3R-NaZrS_2_, where 100 is systematically absent) and in particular the emergence of a strongly broadened reflection that moves from the 102 reflection (1T-ZrS_2_) to the position of the 10–8 in 3R-NaZrS_2_. A schematic representation of the phase transition from 1T-ZrS_2_ to 3R-NaZrS_2_ is shown in Figure 8b.

**Figure 8:**
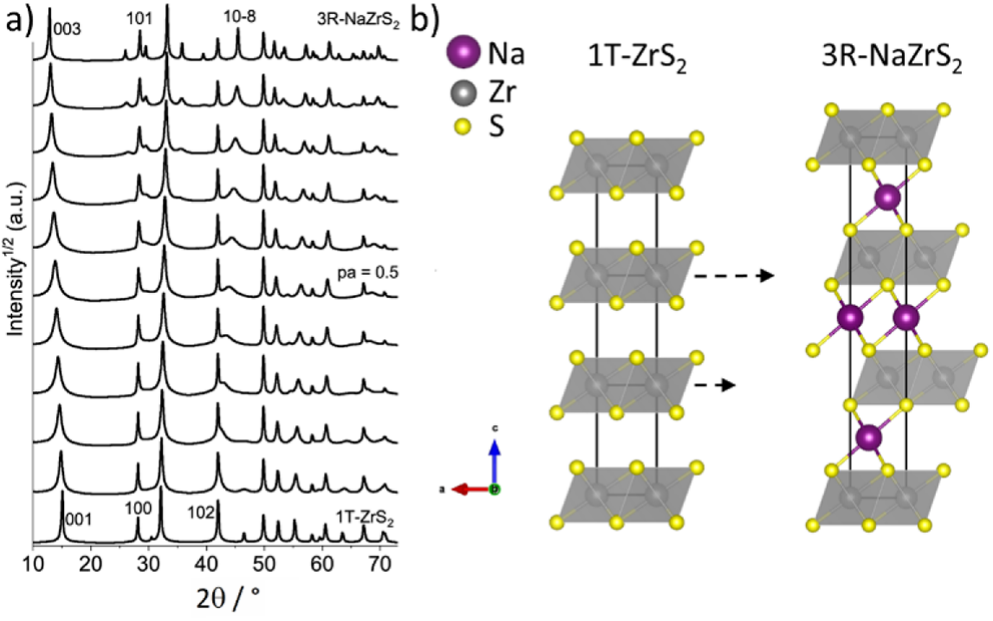
(a) Simulated XRPD patterns for the continuous transition from 1T-ZrS_2_ to 3R-NaZrS_2_. The calculation steps are +0.1 for the fault probability pa each, selected Miller indices are shown and (b) schematic for the phase transformation from 1T-ZrS_2_ to 3R-NaZrS_2_, arrows indicate shifting of the layers.

Using this general approach one can proceed with modeling of the experimental patterns and more detailed questions. First, we studied the effect of Na^+^ intercalation on the interlayer distance for low degrees of intercalation, asking if the *d*-spacing is constant or different from that observed for fully intercalated NaZrS_2_. This was analyzed using the pattern of the sample obtained after 10 mA h g^–1^ discharge (0.06 Na^+^/fu.). From the shift of the 001 reflection an expansion along the *c*-axis is evident, but this could be caused by a low fault probability and a high interlayer distance for the faulted layers or a higher probability combined with a lower interlayer distance (compared to *c* of pure ZrS_2_). The maximum value for *c* would be expected near that of pure NaZrS_2_, which was refined from a sample obtained from high temperature synthesis (*c* = 20.47728(7) Å; *h* = 6.8257(6) Å). Bette et *al.* demonstrated a practical implementation of multidimensional grid searches for such problems in TOPAS.^55^ Such an approach was applied here to optimize the fault probability pa and the layer height for the faulted layers with a fixed layer height from the bulk material when 1T-ZrS_2_ type stacking is present. From the results (see Figure 9b) a clear area with a minimized *r*_wp_ is evident, with 6.85 Å interlayer distance for 3R type stacking and pa = 0.25 being the optimum. As evident from the graph several other combinations nearby yield a similar *r*_wp_, but the result is clear: Once a layer switches to 3R type stacking by intercalation of Na^+^ the interlayer distance takes a value virtually identical to that of NaZrS_2_. Further it is noteworthy, that such a low degree of intercalation of Na^+^ is already sufficient to yield a high number of stacking faults, i.e., an uptake of ∼6% of Na^+^ causes roughly 25% of the layers to shift.

**Figure 9:**
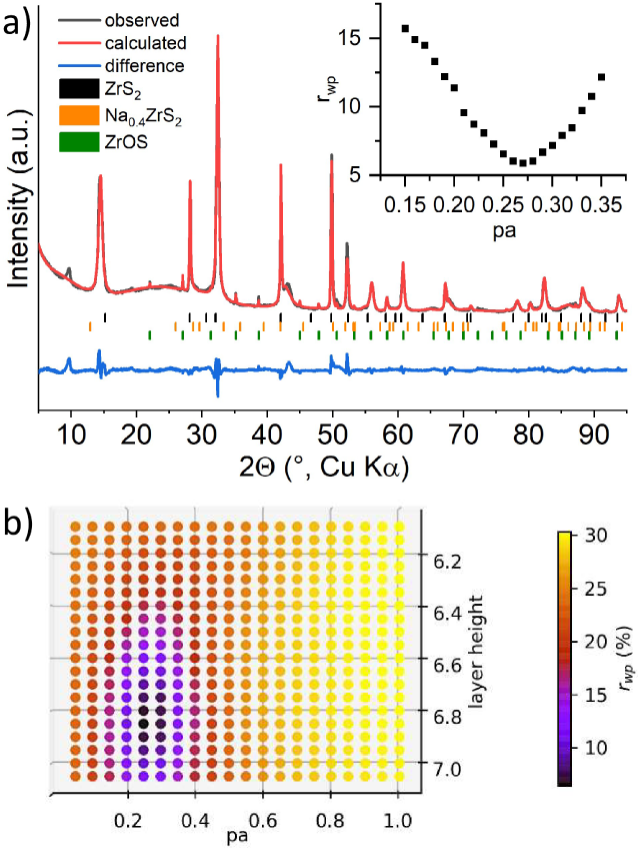
(a) Final difference plot for the sample after uptake of 0.06 Na^+^ per fu. using EC:DEC as electrolyte solvent with an optimized fault probability of 0.27. *r*_wp_ = 4.87%, gof = 3.2, ZrOS 2.2 wt %. Note that the reflection at 9.7° 2θ cannot be described with this model. The inset shows the optimization of the fault probability in smaller steps with refinement of the layer heights. (b) Grid optimization of the fault probability and layer height of the faulted (Na_x_ZrS_2_ type) layers against the powder pattern obtained after intercalating 0.06 Na^+^/f.u.

A further optimization of the fault probability in steps of 0.01 was run with unconstrained refinement of the layer heights in each run, with predefined starting values in each run (6.85 and 5.85 Å). The result (inset in Figure 9a) is a minimum of pa = 0.27, where adjacent values (0.26–0.28) yield virtually identical results in terms of *r*_wp_. Using this value, a Rietveld-refinement of the remaining parameters (zero-point error, layer heights, ...) was carried out. It should be noted that due to absorption problems an empirical correction was carried out until meaningful displacement parameters were obtained. The occupancy of Na^+^ was refined but one should keep the correlation among these parameters in mind when discussing the results. Note also that the occupancy given in Table 1 refers to those layers of type 3R-Na_*x*_ZrS_2_, i.e., occupancy in the entire sample is lower. The final difference plot shown in Figure 9a is satisfactory. The reflection at 9.7° 2θ cannot be explained, its origin remains elusive, its *d*-spacing is not a multiple of the other reflections, i.e., it is not related to a superstructure or staging phenomenon. Otherwise, the faulted structure is in good agreement with the observed diffraction pattern, evidencing that the formation of 3R-type stacking with increased layer distance due to the intercalation of Na^+^ explains the observed changes in the diffraction pattern.

Now we proceed to the data obtained in the compositional range of 0.2–0.35 Na^+^ per fu. The asymmetry of the 001 reflection indicates the formation of either phases with distinct *d*-spacings or domains with differing fault probabilities. This is further corroborated by the shape of the severely broadened reflection in the range of 40–45 °2θ. Note that the shape of this reflection in the simulated diffraction patterns (Figure 8a) is symmetric in contrast to the observed ones. Thus, this requires a slightly modified approach, which is exemplified for the sample with an uptake of 0.3 Na^+^ per fu. First the fault probability was determined by a linear optimization of this parameter and interlayer distances taken from the sample discussed before. For each refinement the parameters were set to those values and allowed to refine. After a first run checking the full range of fault probabilities in steps of pa +0.05 a second run with smaller steps (pa +0.01) was carried out. A minimum in *r*_wp_ (7.5%) at pa = 0.67 was found with similar *r*_wp_ in the range for pa = 0.66–0.69 (Figure 10a). With this value a Rietveld refinement was carried out, the difference plot (Figure 10c) shows—despite the fairly good agreement—two distinct differences that require attention: the split 001 reflection is not matched and the broadened cross plane reflection at ∼45 °2θ is modeled as one broad reflection but the powder pattern actually shows two distinct broad features.

**Figure 10:**
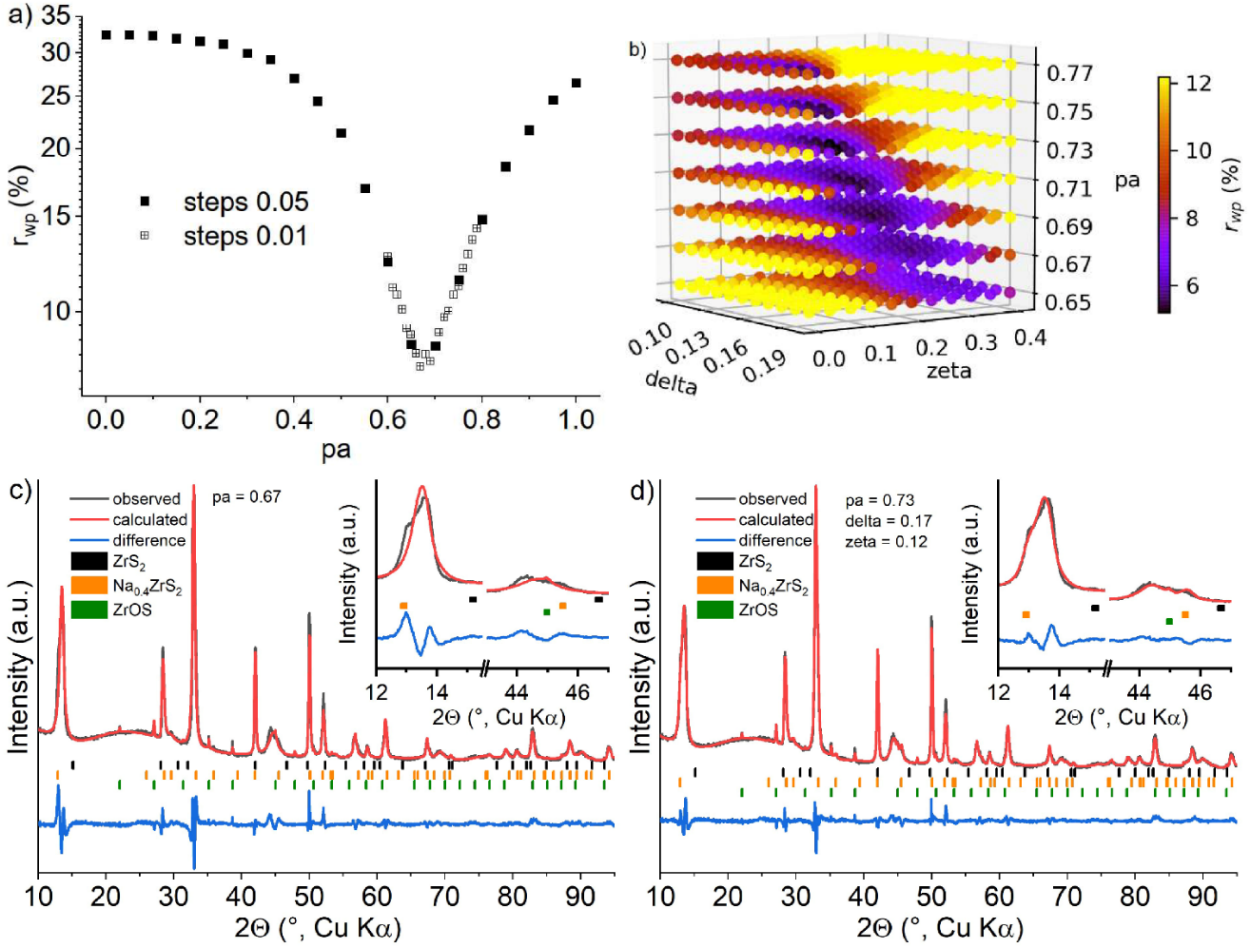
(a) Linear optimization of the fault probability of the sample with 0.3 Na^+^ per fu. intercalated. For the optimum pa the Rietveld refinement is shown (*r*_wp_ = 7.5%) in (c). The result for the three-dimensional grid search on the extended structure model is shown in (b), along with the final Rietveld plot in (d) using the optimum values (*r*_wp_ = 5.0%).

Thus, the model had to be adjusted: In a first attempt two stacking faulted phases with distinct fault probabilities and individual layer distances for the Na_*x*_ZrS_2_ layers were used. The fit improves markedly with *r*_wp_ = 5.6% (Figure S10), the two phases have significantly differing fault probabilities of 0.875 and 0.625 and phase fractions of 29 and 68.5 wt %, which is in line with the less intense shoulder of the 00*l* reflection pointing to lower angles (due to the larger average *d*-spacing) and that of the cross plane reflection around 44–46° 2Θ extending to higher angles, approaching the reflection position of Na_0.4_ZrS_2_. The reflection position of the latter is used as reference, since the crystal structure becomes virtually unfaulted at this composition (cf. Figure 6b)).

We found a somewhat more extended model, that is able to describe the observed patterns even better. In order to account for potential local variations in *d*-spacings or degree of intercalation (possibly due to kinetic hindrance) two different domain types of Na_*x*_ZrS_2_ were introduced in a single faulted phase. They are structurally identical but have a different probability to return to a 1T-ZrS_2_ type stacking. The average fault probability (pa, here 0.67) was kept constant and a parameter called delta controls these two probabilities to be pa ± delta. Further, the probability for faults from a 1T-ZrS_2_ type layer to one of the 3R-Na_*x*_ZrS_2_ domain types (and thus weight of the domains) is controlled via a parameter called zeta, which can vary between 0 and 1 and corresponds to a distribution in pa to yield different probabilities to reach the different domains. A scheme illustrating the two different approaches is shown in Figure S11, a matrix with all transition probabilities for this faulting scenario is given in Table S3. Also the layer distance for the Na_*x*_ZrS_2_ domains was refined in each call with the starting value of 6.85 Å being reset every run. Since the interplay of delta and zeta can change the overall fault probability away from the average value pa and also since the pa from the linear optimization is only a first estimate this parameter was also included in the optimization, such that a total of 3 parameters are included in the grid search.

Two runs were carried out, the first one with coarse steps over the full range for zeta and delta, the second one with finer steps near the optimum of the first run. The resulting 3D plot of *r*_wp_ as a function of the parameters is shown in Figure 10b). The optimum is moved toward larger pa (0.73 instead of 0.67), which is counterbalanced by zeta (0.12), giving large weight to the domains of Na_*x*_ZrS_2_ with a lower probability (pa-delta, i.e., 0.73–0.17) to remain Na_*x*_ZrS_2_ type. Thus, only a small fraction of domains remains with a higher probability Na_*x*_ZrS_2_ type. This is in line with the qualitative observation, that the first 00*l* reflection has a less intense shoulder toward larger *d*-spacings. The final Rietveld fit for this structural model (Figure 10d) shows a significant improvement, both in terms of visual improvement of the fit but also in mathematical agreement reflected by a drop in *r*_wp_ from 7.5% to 5.0% for the simple and extended model. The use of distinct interlayer distances for the two domains of Na_*x*_ZrS_2_ can be justified, as a uniform value results in a significantly higher *r*_wp_ of 5.4%. Note that there are several solutions from the grid search with a similar *r*_wp_, but they all fall in a similar close range of the three parameters. A more detailed discussion of this question can be found in the SI. The extended model yields an even better *r*_wp_ (5.0 vs 5.6%) compared to using two weighted phases with individual stacking fault probabilities, in particular the cross plane reflection around 44–46° 2Θ but also the first split 00*l* reflection are modeled better. This improvement on the line shape may stem from an alternation of long and short domains of Na_*x*_ZrS_2_ being actually present in the sample, which is reflected in the more extended model. Still, both models highly agree on the outcome that the sample is comprised of domains with different fault probabilities and fractions that they contribute with. The difference lies in the implementation of the model and an intergrowth vs a simple summation of two phases.

This approach works equally well on the samples with 0.2 and 0.35 Na^+^ per fu., the plots are shown in panels S12 and S13. From the intercalation of 0.4 Na^+^ per f.u. onward the patterns can be refined with the structure of 3R-Na_*x*_ZrS_2_. All results from the refinements are collected in Table 1. Now we proceed to interpret the results obtained with these extensive refinements, in Tables S4–6 the transition probabilities are listed for the three samples where this extensive approach was applied. First, the global fault probability pa and also occupancy on the site of Na^+^ (despite the correlation with the absorption correction) increase during the gradual crossover from 1T-ZrS_2_ to 3R-Na_*x*_ZrS_2_. These changes are in line with expectations. Lattice parameter *a* undergoes only little changes with very similar values for both phases at the boundary (3.6596(2) Å), while there is a small but significant contraction of up to 0.02 Å as the intercalation proceeds. For 1T-ZrS_2_ type layers the interlayer distance appears to be slightly compressed during intercalation compared to the starting value (5.8243(5) Å). For the 3R-Na_*x*_ZrS_2_ type domains (and later samples) some interesting observations can be made: For the fully intercalated sample we get *h* ∼ 6.80 Å, which is smaller compared to those samples with 0.4 Na^+^/fu. and more, coinciding with the intermediate contraction of *a*. This is also observed for those samples containing large amounts of stacking faults when the transition between the two structure types proceeds. For three of these samples the model with two distinct domains of Na_*x*_ZrS_2_ was assumed with distinct interlayer distances. These differ initially by 0.1 Å and approach each other during further intercalation. The shorter domains (i.e., lower probability to remain 3R-Na_*x*_ZrS_2_, pa – delta) systematically have a larger interlayer distance and these are formed much more frequently (governed by the low value of zeta). This may either be caused by a local pile up of Na^+^ or due to local strain effects. One should bear in mind that within a crystallite probably not each layer is either intercalated to a certain extend or not. The domain model by Daumas and Hérold^56^ suggests that all layers are intercalated to a similar extend but that locally the intercalant accumulates to form here Na^+^ rich regions. The layers would have to adjust by bending and local strain will occur, maybe this results in the excess interlayer distance. The domain model would also explain the sudden lock in in the 3R-Na_*x*_ZrS_2_ structure type when proceeding from 0.35 to 0.4 Na^+^ per f.u. Regarding the absolute values of zeta and delta there is no uniform trend, except that zeta systematically takes values of 0.12–0.22. This can be regarded that always a large number of short Na_*x*_ZrS_2_ and few extended domains of this type exist. To summarize the transition probabilities among the layers (Tables S3–5) calculated from pa, zeta and delta one can state that across the three samples with 0.2–0.35 fu. Na^+^ the domains of ZrS_2_ become less in number and shorter, due to the increasing probabilities to transition to a Na_*x*_ZrS_2_ layer. The shorter of those domains (indicated as “low” due to lower probability to persist) are formed more frequently (higher transition probability from ZrS_2_ type layer) compared to the more extended ones (on average ∼60% vs ∼15% probability). Both become systematically more extended with increasing Na^+^ content. Further we note that the errors for zeta (governing the probability to transition from ZrS_2_ layers to one of the Na_*x*_ZrS_2_ domains) gives no clear trend and comes with fairly large errors (estimated from grid points with similar low *r*_wp_, see Table 1). Still, all other resulting fault probabilities (5 out of 7 total) give the consistent trend outlined above, showing that the model works well overall and reflects the qualitative observations in widely reasonable numbers.

**Table 1:** Collection of All Structural Parameters of the *Ex Situ* Samples Collected during the First Discharge of ZrS_2_ against Na Metal^a^

ID	*a*/Å	h (ZrS_2_)/Å	h (Na_*x*_ZrS_2_)/Å pa – delta	h (Na_*x*_ZrS_2_)/Å pa + delta	pa	zeta	delta	occ (Na) for Na_*x*_ZrS_2_ layers, faulted phase
0	3.6596(2)	5.8243(5)	-	-	-	-	-	-
0.06	3.6553(1)	5.8291(7)	6.824(1)		0.27(1)	-	-	0.22(3), 0.06(1)
0.2	3.6467(1)	5.782(2)	6.906(1)	6.801(1)	0.67(0)	0.22(2)	0.14(1)	0.39(1), 0.26(1)
0.3	3.6457(1)	5.798(2)	6.884(1)	6.829(1)	0.73(2)	0.12(4)	0.17(1)	0.437(9), 0.31(1)
0.35	3.6427(1)	5.791(3)	6.884(1)	6.8431(5)	0.82(1)	0.22(4)	0.12(1)	0.429(9), 0.36(5)
0.40	3.6406(5)	-	6.8530(2)		-	-	-	0.43(1)
0.55	3.64184(3)	-	6.8460(1)		-	-	-	0.57(1)
0.8	3.65858(3)	-	6.8084(1)		-	-	-	0.88(1)
0.99	3.66409(3)	-	6.7944(1)		-	-	-	0.94(1)

a For h (Na_*x*_ZrS_2_) the index +/– refers to those domains where delta is added to/subtracted from pa, respectively. The error values are standard deviations based on the five solutions with lowest *r*_wp_ from the corresponding grid search. The occupancies for Na are given in values for the site occupancy in the layers of Na_*x*_ZrS_2_ type as well as product of this value and pa as total occupancy in the faulted phase.

The occupancy for sodium in the crystal structure (Table 1) varies systematically across the samples, independent of the used structure model (ideal or faulted structures) and agrees well with the nominal compositions. For the faulted models both the site occupancy factor in the Na_*x*_ZrS_2_ layers is given as well as the composition compared to Zr for the entire phase, which is lower as it is “diluted” by the ZrS_2_ layers. The latter value is straightforward to be compared with the nominal composition.

### *In Situ* X-ray Diffraction

Since the sampling of the (dis-)charge states via *ex situ* measurements might bring about structural changes, e.g., due to relaxation, an *in situ* XRPD study was performed covering the first four and a half cycles. The voltage profile is similar to the previously observed profiles from the *ex situ* cycling experiments (Figure 6b). Initially, the expected 1T-ZrS_2_ phase was observed. By lowering the voltage and starting the intercalation reaction a reflection shift is observed until the end of the plateau in the voltage profile (Figure 11a,b). Namely, the 001 reflection shifts from 5.4 (*d* = 6.0 Å) to 4.6° 2θ (*d* = 7.0 Å) (note that this experiment was performed with Ag–Kα_1_ radiation) as expected for an increasing interlayer spacing while Na^+^ is intercalated (Figure 11a). Simultaneously, the 100 → 101 and 101 → 104 reflections shift contrarily from 10.40° (*d* = 3.09 Å) to 10.48° 2θ (*d* = 3.07 Å) and 11.5 (*d* = 2.80 Å) to 12.0° 2θ (*d* = 2.68 Å), respectively. Further the formation of the 018 reflection at 16.2° 2θ (*d* = 1.99 Å) from 3R-NaZrS_2_ can be observed (Figure 11a,d). At a potential of 1.4 V (+0.6 Na^+^/fu.) only reflections which can be assigned to the 3R-Na_*x*_ZrS_2_ phase were observed. Within the last few hours of discharge only slight shifts of reflections at 17.5°, 18.3° and 21.4° 2θ can be observed. The reversible phase transition of 3R-NaZrS_2_ to 1T-ZrS_2_ only takes place within the last two hours of charging. The reflections shift abruptly to smaller diffraction angles when the potential increases after the voltage plateau. Sequential Rietveld refinement of the region from 10.5 to 25 h of cycling, i.e., end of discharge until mid of charge, shows a correlation between the increase of the cell volume and the occupancy of Na^+^ ions. Both values are maximized at the end of the discharge (Figures S14, 18h). Recharging the cell leads to a decreasing cell volume in conjunction with the release of Na^+^ ions. By further release of Na^+^ ions (0.8–1 Na^+^/fu.) the reflections shift to their initial positions and the 1T-ZrS_2_ structure is retrieved (Figure 11a,c). For the following cycles a similar reflection shift is observed proving the reversibility of the intercalation reaction. Even after multiple cycles the reflection shift can be observed in detail (Figure 11c,d). *Ex situ* XRPD measurements from the first cycle showed similar reflection shifts within the discharge but what we have learned from the *in situ* XRPD study is the abrupt phase transition of 3R-NaZrS_2_ to 1T-ZrS_2_ in the charge and the evidence for the reversibility of the reaction with regression of the initial 1T-ZrS_2_ phase.

The same measurements were performed for ZrS_2_ vs Na^+^|Na using NaOTf in diglyme as electrolyte. The results are shown in Figures S15–S16. However, in this experiment the behavior observed in the *ex situ* experiments described above could not be reproduced. The starting compound is also 1T-ZrS_2_ but contrarily to the measurements using the electrolyte NaTFSI in EC:DEC the reaction pathway cannot clearly be followed. Mainly, information about a large volume expansion along the *c*-axis can be concluded which is indicated by the disappearance of the 001 reflection at 5.5° 2θ and the appearance of a new reflection at 4.5° 2θ (Figure S16a). This is the formation of a cointercalated Na–diglyme Na_*x*_ZrS_2_ phase. Further, cross plane reflections disappear after the first discharge and either appear at lower diffraction angles (100, 101/011, 110; Figure S16b,c) or they reappear at a later state of cyclization. However, it is striking that neither the formation of 3R-Na_*x*_ZrS_2_ nor the reaction back to 1T-ZrS_2_ can be observed. In the voltage profile the new positioning of the reflections corresponds to the first change of the slope in the first discharge. In the following cycles the voltage profile shows no significant changes which indicates a full conversion within the first discharge.

**Figure 11:**
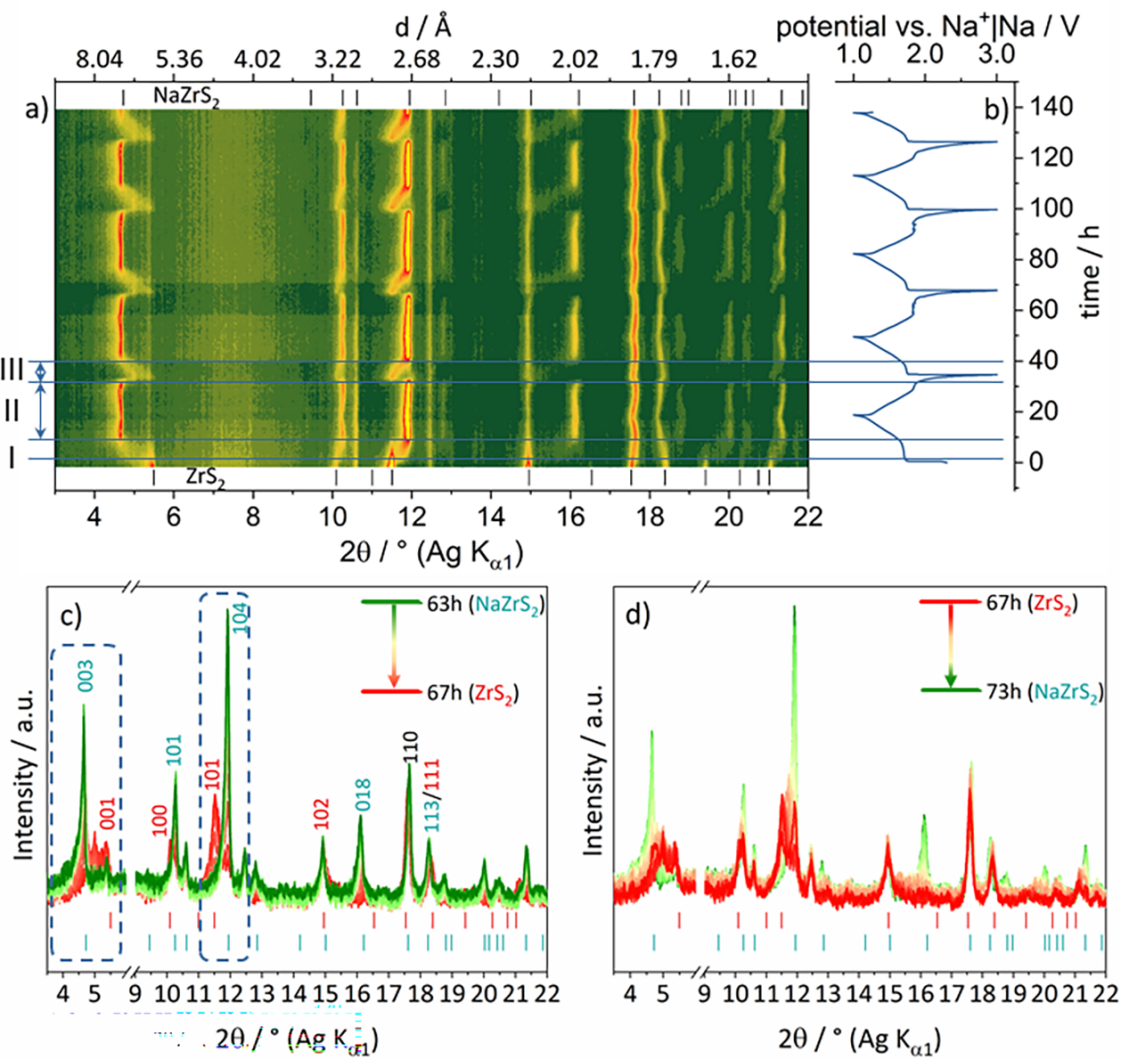
(a) Contour plot of *operando* diffraction pattern with the corresponding voltage profile (b) using ZrS_2_ electrodes cycled in NaTFSI in EC:DEC vs Na^+^|Na. The theoretical patterns of ZrS_2_ and NaZrS_2_ are shown as ticks. Highlighted are the different regions of ZrS_2_ (I), NaZrS_2_ (II) and the phase transition of NaZrS_2_ → ZrS_2_ → NaZrS_2_ (III). (c) Detailed 2D plots of the reflection shifts during the recharge within the second cycle (after 63–67 h of cycling). Dashed boxes show the reflection shift of the most pronounced reflections (001 → 003; 101 → 104). (d) the third discharge (after 67–73 h of cycling). *hkl* indices belonging to ZrS_2_ are red colored and *hkl* indices of NaZrS_2_ are green colored. The 110 reflection belongs to both, ZrS_2_ and NaZrS_2_, and is black colored.

### Solid State ^23^Na Nuclear Magnetic Resonance Spectroscopy

We performed *ex situ* ^23^Na MAS NMR spectroscopy to analyze whether different species of Na^+^ ions during the intercalation into the host structure of ZrS_2_ can be identified. Multiple peaks in the NMR spectra might indicate different crystallographic sites in one or multiple phases, but also Na ions located on the same site but with different environments, i.e., anion/cation neighbors. According to the observed results of *ex situ* XRPD the NMR spectra of samples from diglyme containing cells show peaks different from the equivalent cells cycled with EC:DEC. As expected, the pristine sample is sodium-free causing no observable peaks in the NMR spectrum. In total four different peak shifts are observed for diglyme based cells (Figure 12a). For all samples a peak at −14 ppm can be observed matching the reference measurement of the conduction salt NaOTf. This peak can thus be assigned to the residues of the electrolyte salt. A peak with increasing intensity (9 ppm) is observed for samples after an uptake of 0.2–0.6 Na^+^/fu. which indicates the formation of a sodium species at the beginning of the second plateau in the voltage profile, further intercalation of sodium (0.8 Na^+^/fu.) and a release of 0.3 and 0.6 Na^+^/fu. leads to decreasing intensity of this peak. The same samples show a peak of low intensity at 21 ppm as well as a broadened peak at 79 ppm (+0.2 Na^+^/fu.) which shifts to 91 ppm (+0.8 Na^+^/fu.). While narrow peaks indicate highly symmetric coordination, broad peaks are observed for unsymmetric coordination or sluggish kinetics. Thus, the peak at 9 ppm may be assigned to Na–diglyme complexes which are adsorbed at the electrode surface. The peak at 21 ppm can be assigned to highly symmetric coordinated Na^+^ in the interlayer spaces. A reference measurement of NaZrS_2_ obtained from high temperature synthesis shows a broad peak at 96.7 ppm (Figures 12 and S17). Therefore, peaks shifting from 79 to 91 ppm during the intercalation of Na^+^ and the shifting back to 81 ppm during the deintercalation of Na^+^ are assigned to the formation of Na_*x*_ZrS_2_ (*x* = 0.2, 0.5, 0.6, 0.7, 0.8), e.g., asymmetrically coordinated Na^+^ in the interlayer space.

**Figure 12:**
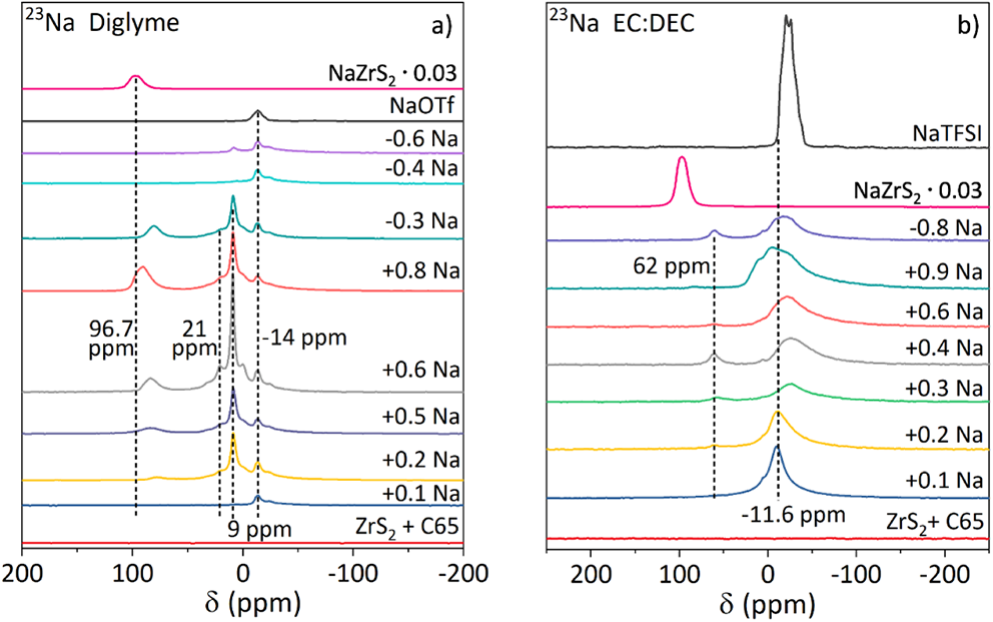
^23^Na MAS NMR spectra of ZrS_2_ for different intercalation states using a diglyme based electrolyte (a) and an ethylene carbonate/diethyl carbonate based electrolyte (b).

In contrast the NMR spectra observed for different Na^+^ uptake using a carbonate based (i.e., EC:DEC) electrolyte show two pronounced peaks (Figure 12b). One peak can be observed at −10 ppm which is symmetric for the sample after an uptake of 0.1 Na^+^/fu. and becomes more asymmetric for an uptake of 0.2 Na^+^/fu. and shifts to −26 ppm (0.3 Na^+^/fu.), −25 ppm (0.4 Na^+^/fu.) and −22 ppm (0.6 Na^+^/fu.). For an uptake of 0.9 Na^+^/fu. the peak remains at −10 ppm but an asymmetric shape as well as a shoulder at 10 ppm were observed. This peak is a combination of the conducting salt NaTFSI (−11.6 ppm) and may also result from adsorbed electrolyte on the electrode surface. After the release of 0.8 Na^+^/fu. an asymmetric and broadened peak at −17 ppm is observed and a shoulder at 6 ppm. There is a second peak at 61 ppm which shows the highest intensity after an uptake of 0.4 Na^+^/fu. and the release of 0.8 Na^+^/fu., respectively. A detailed view of the chemical shift between 0 and 150 ppm is shown in Figure S17 for those samples where this feature is much lower in intensity. A less pronounced peak is observed at 61 ppm for the sample after an uptake of 0.2 Na^+^/fu. This peak shifts with increasing uptake of Na^+^ to 84 ppm (+0.9 Na^+^/fu.). Comparable to the cells cycled with the diglyme based electrolyte, the shift of the peaks with increasing degree of intercalation can be compared with the reference obtained from high temperature synthesis (96.7 ppm) and it can be concluded that intercalated Na^+^ is present in the interlayer spaces. In the voltage profile (Figure 6) the discharge plateau ends at an uptake of 0.4 Na^+^/fu. and the charge plateau ends at a release of 0.8 Na^+^/fu.

### Electrochemical Cycling and Effect on the Structure

To determine the electrochemical properties of ZrS_2_ as electrode material vs Na^+^|Na the cycle stability and the structural changes after the first 5 cycles were investigated. Therefore, circular film electrodes were cycled in two different potential ranges (0.3–2.8 V; 1–2.8 V) using the electrolyte solvents EC:DEC (1:1), diglyme, THF and PC. The additional electrolyte solvents were used to prove the influence of strongly and weakly coordinating solvents. The higher cutoff voltage was applied to avoid side reactions, which are unavoidable at low potentials, and check for effects on the cycling stability. Cycling ZrS_2_ vs Na^+^|Na in EC:DEC in a potential range of 0.3–2.8 V leads to an initial specific capacity of 255 mA h g^–1^ which is equal to an uptake of 1.5 Na^+^/fu. (Figure 13a). While the potential plateau and the following decreasing potential until 200 mA h g^–1^ indicate an intercalation reaction and the shape of the discharge curve between 200 and 255 mA h g^–1^ indicates a beginning conversion reaction. Furthermore, the maximum uptake of 1.5 Na^+^/fu. in the first discharge can be caused by the formation of a solid electrolyte interphase (SEI). The initial discharge curve shows a plateau at 1.6 V until a specific capacity of 88 mA h g^–1^ is reached and the phase transition from 1T-ZrS_2_ to 3R-Na_*x*_ZrS_2_ is completed. Afterward, a slight decrease in the potential until 1.1 V (185 mA h g^–1^) and a large potential drop until 0.3 V (255 mA h g^–1^) are observed. The following cycles show similar behavior apart from the intercalation plateau extending over less capacity. This is accompanied by a decreasing specific capacity down to 150 mA h g^–1^ in the fifth discharge.

**Figure 13:**
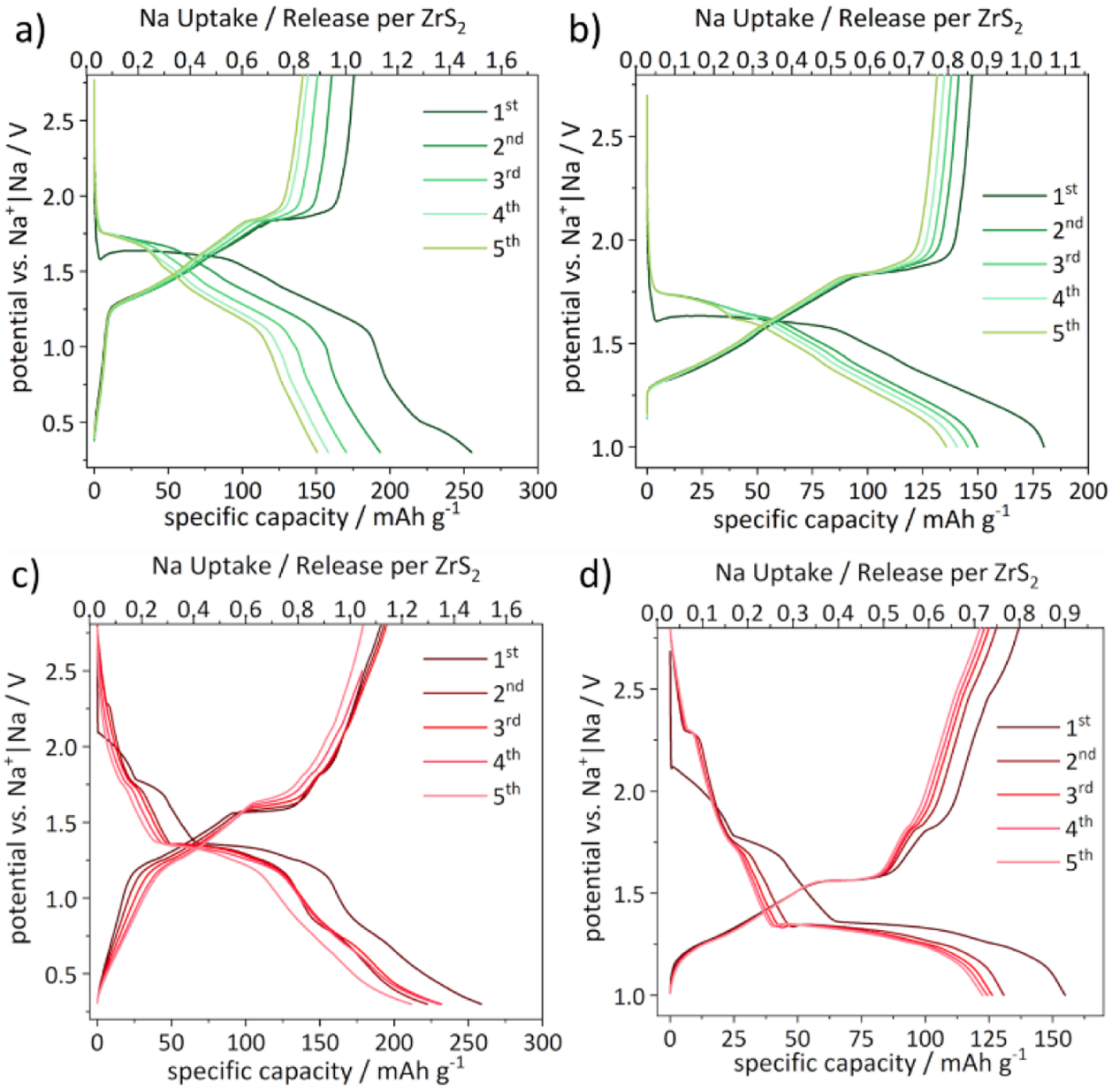
Voltage profiles of test cells cycled with an EC:DEC-based (a and b, green) and a diglyme-based (c and d, red) electrolyte in a potential range of 0.3–2.8 V and 1–2.8 V, respectively.

Now the evolution of the charge process is discussed: During the first charge a specific capacity of 175 mA h g^–1^ could be obtained which equals a release of 1 Na^+^/fu. Between 0.3 and 1.25 V a large increase of the potential but not in the specific capacity (only 12 mA h g^–1^) is observable. Until 1.8 V the specific capacity increases gradually to 116.5 mA h g^–1^ and afterward a plateau is observed until a specific capacity of 160 mA h g^–1^ where the reverse phase transition from 3R-Na_*x*_ZrS_2_ to 1T-ZrS_2_ likely takes place. From 1.8 to 2.8 V a fast increase of the potential and only a slight increase of the specific capacity to 175 mA h g^–1^ can be observed. Also the following cycles show similar behavior but with a decreasing length of the plateau which corresponds to a decreasing capacity until 141 mA h g^–1^ after the fifth charge (Figure 13a) are obtained. By further cyclization the capacity fades quickly until a specific capacity of 83.5 mA h g^–1^ within the first 20 cycles is measured. Afterward, the capacity fades slowly but constantly to a specific capacity of 55 mA h g^–1^ after the 500^th^ cycle (Figure S18).

To reduce the stress on the material or avoid a conversion and other side reactions the lower cutoff voltage was increased to 1 V instead of 0.3 V. This potential corresponds to ∼1 Na^+^/fu. and also marks the onset of the next potential drop after the presumably full intercalation of ZrS_2_ to NaZrS_2_. Thereby, the cycle stability could be increased. After the fifth cycle the capacity loss was 22% smaller than in the large potential range (Figure 13b). However, no significant improvement of the cycle stability can be observed by further cycling over 500 cycles (Figure S18).

In contrast, ZrS_2_ vs Na^+^|Na cells cycled with diglyme as electrolyte solvent show a different behavior of the potential vs specific capacity (Figure 13c,d). Within the first discharge two pseudo plateaus can be observed at 2.1 V–1.9 V (19 mA h g^–1^) and 1.8 V–1.7 V (43 mA h g^–1^) which corresponds to the cointercalation of Na^+^ and diglyme (or a complex thereof) into ZrS_2_. At a potential of 1.4 V a plateau can be observed from a specific capacity of 66 mA h g^–1^ until 137 mA h g^–1^ are reached which can be assigned to the coexistence of Na_*x*_ZrS_2_ structures with different interlayer distances. Afterward, the potential decreases rapidly until a specific capacity of 259 mA h g^–1^ is reached at 0.3 V which corresponds to an uptake of 1.5 Na^+^/fu. In the following cycles the two pseudo plateaus disappear which leads to an earlier start of the extended plateau at a specific capacity of 46 mA h g^–1^ resulting also in a decrease of the specific capacity after the full discharge of the cell. In the second until the fourth cycle a reversible specific capacity of 230 mA h g^–1^ is observed and in the fifth cycle the specific capacity drops to 212 mA h g^–1^. The curve of the first charge is similar to the behavior observed for the cells cycled with EC:DEC as electrolyte solvent. A steep increase of the potential with almost no increase of the specific capacity (24 mA h g^–1^) followed by a slightly sloping increase in specific capacity until a plateau appears at a potential of 1.55 V, can be observed. After this plateau, the potential steeply increases until the cut off voltage of 2.8 V, with two small but distinct steps. A specific capacity of 193 mA h g^–1^ which corresponds to an uptake of 1.1 Na^+^/fu. was reversibly achieved until the fourth cycle but the slope and the length of the plateau are changing over the cycle number. This causes a capacity loss in the fifth cycle down to 179 mA h g^–1^ (Figure 12c). Cyclization beyond 500 cycles leads to a significant loss in capacity within the first 50 cycles (until 100 mA h g^–1^). Afterward, the capacity begins to stabilize but still slowly fades until a specific capacity of 50 mA h g^–1^ is reached in the 500^th^ cycle (Figure S19). This loss in capacity can be caused by the incomplete deintercalation of sodium ions during charging and following slowly decomposition of the electrode resulting in disconnection between the current collector and the active material or the grain boundaries of crystals within the electrode. Further, SEM images of the electrodes (Figure S20) show the embedding of ZrS_2_ crystals in conducting carbon. Sodium only reacts with ZrS_2_ causing expansion and contraction of the layered structures which causes inhomogeneities in the electrodes during cycling. The more often the electrode is cycled, the higher is the impact of contact loss between the grain boundaries. Further, an explanation for fast fading capacity could be the cell type used for these experiments. Since the self-made Swagelock type test cells are originally not designed for long-term measurements and have a lower degree of sealing as well as the pressure exerted inside the cell cannot be adjusted. That could also explain the differences between the cycle stability tests and the cycle performance of coin cells used for *in situ* XRPD measurements.

The cutoff voltage was also increased to 1 V for the cells that were cycled with diglyme as electrolyte solvent to decrease the stress on the material. Here, the two pseudo plateaus, which are less distinct, were also obtained in the second to fifth discharge where the first pseudo plateau shifts to a higher potential of 2.3 V. During the charge process, the curves are similar until the end of the plateau at a potential of 1.55 V. A capacity loss from the first to the second cycle was observed but from the second to the fifth cycle the specific capacity is almost constant (Figure 13d). Overall, the impact on the long-term stability using a higher cutoff voltage appears to be less pronounced for cells containing diglyme as electrolyte solvent compared to EC:DEC. Nevertheless, cycle stability can be increased by lowering the cutoff voltage to 1.1 V (Figure S19).

As a further extension, cycling experiments were also performed with the noncoordinating solvent THF and the coordinating solvent PC. Cells cycled with THF as electrolyte solvent show a similar behavior to the cells containing EC:DEC as electrolyte solvent with the difference that the plateau appears at a higher potential of 1.75 V in the discharge and at 1.85 V in the charge compared to 1.6 and 1.75 V for EC:DEC (Figure S21a/b). Cells cycled with PC as electrolyte solvent show a different behavior compared to all other cells. In the wide potential range between 0.3 V–2.8 V the highest specific capacity of 340 mA h g^–1^ in the first discharge was obtained by using PC as electrolyte solvent. This corresponds to about twice the theoretically expected capacity and indicates pronounced side reactions in the electrochemical cell. This is also indicated by the significant loss of capacity in the subsequent cycles, which is already 49% in the second cycle and even more in the following cycles. In addition, the course of the curve differs from the other electrolytes. At the beginning of a plateau at 1.6 V the potential increases reproducible to 1.8 V (7 mA h g^–1^) and drops in the following until a specific capacity of 10 mA h g^–1^ is reached. After the plateau at 68 mA h g^–1^ in the first discharge a continuous decrease of the potential is observed until the cutoff voltage is reached. The charge is similar to the results observed for the cells cycled with other electrolyte solvents (Figure S21c/d).

To analyze the influence of cycled uptake and release of sodium ions on the structure of ZrS_2_ XRPD measurements were performed after the fifth cycle in the charged state of each cell (Figure 14) for the two different potential windows. For all electrolytes only few sharp reflections were observed. Further, the data can be grouped: The samples obtained with EC:DEC and THF show similar patterns. Sharp reflections are observed at positions corresponding to sodiated Na_*x*_ZrS_2_ and ZrS_2_. Particularly the reflection at 14.1° 2θ (*d* = 6.26 Å) with different *d*-spacing in between the 00*l* reflections for NaZrS_2_ and ZrS_2_ point to an incomplete deintercalation of Na^+^. In contrast, the samples obtained with the strongly coordinating solvents PC and diglyme show only few sharper reflections. In detail the powder patterns of cells cycled with a lower cutoff voltage of 0.3 V using EC:DEC as electrolyte solvent show reflections at 13°, 33.4°, 45.5° 2θ which can be assigned to the NaZrS_2_ phase and reflections at 15.1°, 32.2° and 42.1° 2θ which can be assigned to the ZrS_2_ phase as well as additional reflections at 14.1°, 32.7° and 53.6° 2θ which are supposed to result from an incomplete release of sodium ions in the charge process (Figure 14, dark green). This is in accordance with the potential curves from the cycling experiments because the specific capacity after charging the cells differ from the capacity in the respective discharge, which indicates an incomplete deintercalation of sodium ions. Further a sharp reflection at 6.5° 2θ is observed for samples cycled in the large potential range (0.3–2.8 V) independent from the electrolyte solvent (EC:DEC, diglyme, THF). This reflection cannot be assigned to a known phase. Typical side phases like Na_2_S, NaF or Na_2_O do not fit the position of this reflection. When applying a higher cut off voltage of 1 V the reflection at 6.5° 2θ is not observable. It seems the intensity of the reflections belonging to the NaZrS_2_ phase are of lower relative intensity compared to the reflections assigned to ZrS_2_ compared to the cells cycled in a larger potential range (Figure 14, light green). As already suspected from the cycle measurements, cells cycled with THF as electrolyte solvent show the same reflection positions in the XRPD pattern after five cycles. Likewise, the intensity of the reflections belonging to ZrS_2_ are of higher intensity than the reflections belonging to the sodiated phase (Figure 14, blue). Unlike, reflections of the additional phase are of lower intensity and another additional reflection at 18° 2θ appears for the cells cycled in a potential range from 2.8 to 1 V (Figure 14, light blue). Cells cycled with diglyme as electrolyte solvent show a completely different XRPD pattern after five cycles (Figure 14, red). When applying a lower cutoff voltage of 0.3 V only broadened reflections were observed.

**Figure 14:**
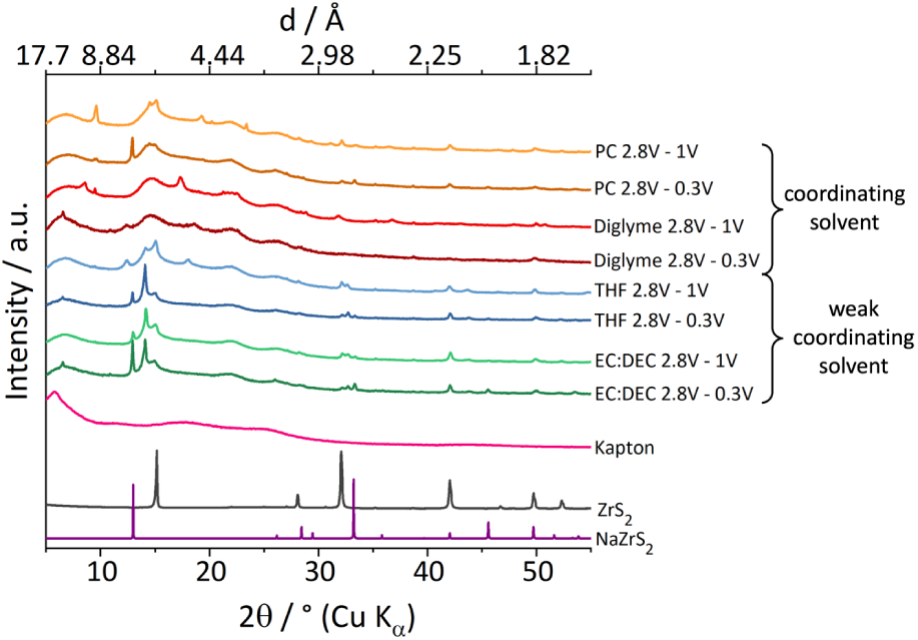
XRPD pattern of ZrS_2_ vs Na^+^|Na in the 5^th^ charged state using different electrolyte solvents as indicated. Kapton foil was used to enclose the samples. The broad reflections of the Kapton match the broad reflections observed in the pattern of the samples after cycling.

The powder pattern of the cells cycled with a lower cutoff voltage of 0.3 V using diglyme as electrolyte solvent show only broadened reflections except for a reflection at 6.5° 2θ which was also observed for the electrodes cycled with THF and EC:DEC as electrolyte solvents (Figure 14, dark red). Increasing the lower cut off voltage to 1 V leads to additional reflections at 8.5°, 9.5°, 17.3° and 36.8° 2θ which cannot be assigned to either NaZrS_2_ or ZrS_2_ (Figure 14, light red). This is in accordance with the results of the *ex situ* investigation which showed a large volume expansion during Na uptake. After the extraction of sodium ions nearly no information of the crystal structure was observed. A comparison can be made with the data from the *ex situ* XRPD measurements, in which a strongly disordered material is already present after the first cycle. The long-range order is further reduced with further cyclization. Results of the XRPD after 5 cycles using PC as electrolyte solvent reveal sharp as well as broadened reflections while the sharp reflection in the XRPD pattern of the cell cycled until the lower cutoff voltage of 0.3 V can be assigned to the NaZrS_2_ phase (Figure 14 dark orange), the reflections in the pattern of the cells cycled until a lower cutoff voltage of 1 V are in good agreement with the ZrS_2_ phase (Figure 14, light orange). Further, there are additional reflections at 9.5°, 19.3° and 23.3° 2θ, which cannot be assigned to a known phase.

In conclusion, cycling of ZrS_2_ vs Na^+^|Na using different electrolytes results in a loss of long-range order and an incomplete release of sodium ions during the charge. Electrolyte solvents which are known to form complexes with sodium ions, such as diglyme and PC, lead to a large volume change during cycling followed by a capacity loss in the following cycles. Further, a multistep potential curve was observed for these cells. In contrast, weakly coordination electrolyte solvents like THF and EC:DEC lead to materials that show a few sharp reflections after cycling. However, these can be equally assigned to fully occupied, partially occupied and unoccupied domains. There are noticeable differences in intensity between the reflections of the fully occupied and unoccupied domains between the cells that were cycled between 1 and 2.8 V and those that were cycled in between 0.3 and 2.8 V. In the smaller potential window, the intensities of the reflections that can be assigned to the unoccupied ZrS_2_ phase are more intense and in the large potential window the reflections of the fully occupied NaZrS_2_ phase and the partially occupied Na_*x*_ZrS_2_ phase are more intense. This shows that the deintercalation of the sodium ions does not take place completely, neither in the large nor in the small potential range. But when applying the smaller potential window, more Na^+^ can be extracted during charge as in the larger potential range. Nevertheless, the cycle stability increases in a decreasing potential range because of the avoidance of side reactions at low potentials and therefore less mechanical stress inside the material.

## Conclusions

In this study we have shown the differences between intercalation pathways by using different electrolytes, providing numerous insights into the Na-based intercalation chemistry of ZrS_2_. Strongly coordinating solvents like diglyme cointercalate into the layered structure of ZrS_2_ which leads to a significant expansion of the interlayer spacing at low uptake of sodium as a consequence. During further intercalation the solvent is deintercalated and the remaining structure can be assigned to heavily stacking faulted 3R-Na_*x*_ZrS_2_. In contrast, using weakly coordinating solvents like EC:DEC leads to an intercalation reaction which causes a phase transition from 1T-ZrS_2_ to 3R-NaZrS_2_. This phase transition was analyzed structurally with the result that successively formed stacking faults lead directly from one type to another, whereby domains of different degrees of intercalation were observed. Samples with high crystallinity were also obtained after the first charge. A detailed analysis of the stacking faults showed the simultaneous intercalation of the layers and the direct expansion to the layer distance of the NaZrS_2_ when the stacking fault occurs. From the results it follows that the intercalation reaction differs from previously studied Na^+^ intercalation into TiS_2_ where more intermediates are observed. For ZrS_2_ there is a direct phase transition from 1T-ZrS_2_ to 3R-NaZrS_2_ induced by a few stacking faults. Long-term cycling experiments result in a loss of long-range order, regardless of the solvent, whereby more crystalline samples are obtained with a narrower potential window and the usage of noncoordinating solvents. Cycling up to 500 cycles leads to similar voltage profiles and specific capacities what indicates a minor role of the solvents with increasing cycle numbers. This indicates that other factors aside from structural integrity or type of solvent become dominant for the long-term retention of capacity and cycling stability like the degree of sealing of the cells, which is rather an engineering than chemistry centered question. Future experiments could be carried out in hermetically sealed cells such as pouch or coin cells.

## Supplementary Material





## Notes

Additional experimental data and materials, including photographs of experimental setup (PDF)
Input files (ZIP)
