# Supplementary material for: Electrochemical Intercalation Reaction of Sodium into the Layered Transition Metal Dichalcogenide ZrS2—Influence of the Electrolyte Solvent
Source: Inorg Chem. 2025 Aug 27;64(36):18045–61. doi: 10.1021/acs.inorgchem.5c02331 (PMC12442063; doi:10.1021/acs.inorgchem.5c02331)
Supplement: Supplementary file 1 [file ic5c02331_si_001.pdf]

Supporting Information

Electrochemical intercalation reaction of sodium into the layered transition metal dichalcogenide ZrS_2 – Influence of the electrolyte solvent

Lina Liers[a], Liuda Mereacre[b], Hang Li[b], Julia Mickenbecker[a], Michael Knapp[b], Sylvio Indris[b,c], Malte Behrens[a], Sebastian Mangelsen*[a]

[a] Institute of Inorganic Chemistry, Kiel University, Max-Eyth-Str. 2, 24118 Kiel, Germany. *Sebastian Mangelsen: smangelsen@ac.uni-kiel.de

[b] Institute for Applied Materials - Energy Storage Systems, Karlsruhe Institute of Technology, P.O. Box 3640, 76021 Karlsruhe, Germany

[c] Applied Chemistry and Engineering Research Centre of Excellence (ACER CoE), Université Mohammed VI Polytechnique (UM6P), Lot 660, Hay Moulay Rachid, Ben Guerir, 43150, Morocco

Supplementary Figures

Sample cell for in-situ XRPD measurements

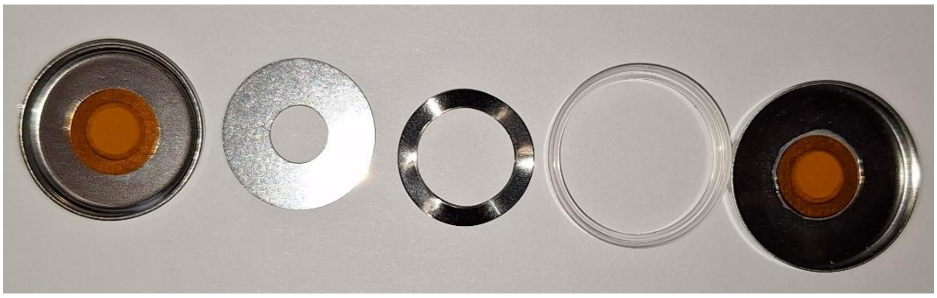

Figure S1: Picture of a handmade coin cell for in-situ XRPD measurements. The individual parts each have a hole in the middle to let the X-ray beam through.

Table S1: Crystallographic and Rietveld refinement data for Na_xZrS_2.

Sample	Na_xZrS_2 (x = 0.82) derived from high temperature synthesis	Na_xZrS_2 (x =0.95) – electrochemical intercalation with EC/DEC electrolyte solvent
CCDC	2470929	2470930
Molecular weight (g/mol)	173.943	177.20
Space group / IT Number	*R* -3 *m* / 166	*R* -3 *m* / 166
Z	3	3
a / Å	3.65647(3)	3.66408(3)
b / Å	3.65647(3)	3.66408(3)
c / Å	20.4772(3)	20.3833(3)
α / Å	90	90
β / Å	90	90
γ / Å	120	120
V / Å³	237.097(5)	236.933(5)
ρ_{calc} / $g \cdot cm^{-3}$	3.66	3.72
Wavelength / Å	1.540598	1.54067
R-exp / %	2.88	1.46
R-wp / %	3.67	3.68
R-Bragg / %	1.56	1.83
R-p / %	2.76	2.78

Scanning electron microscopy of the pristine ZrS_2

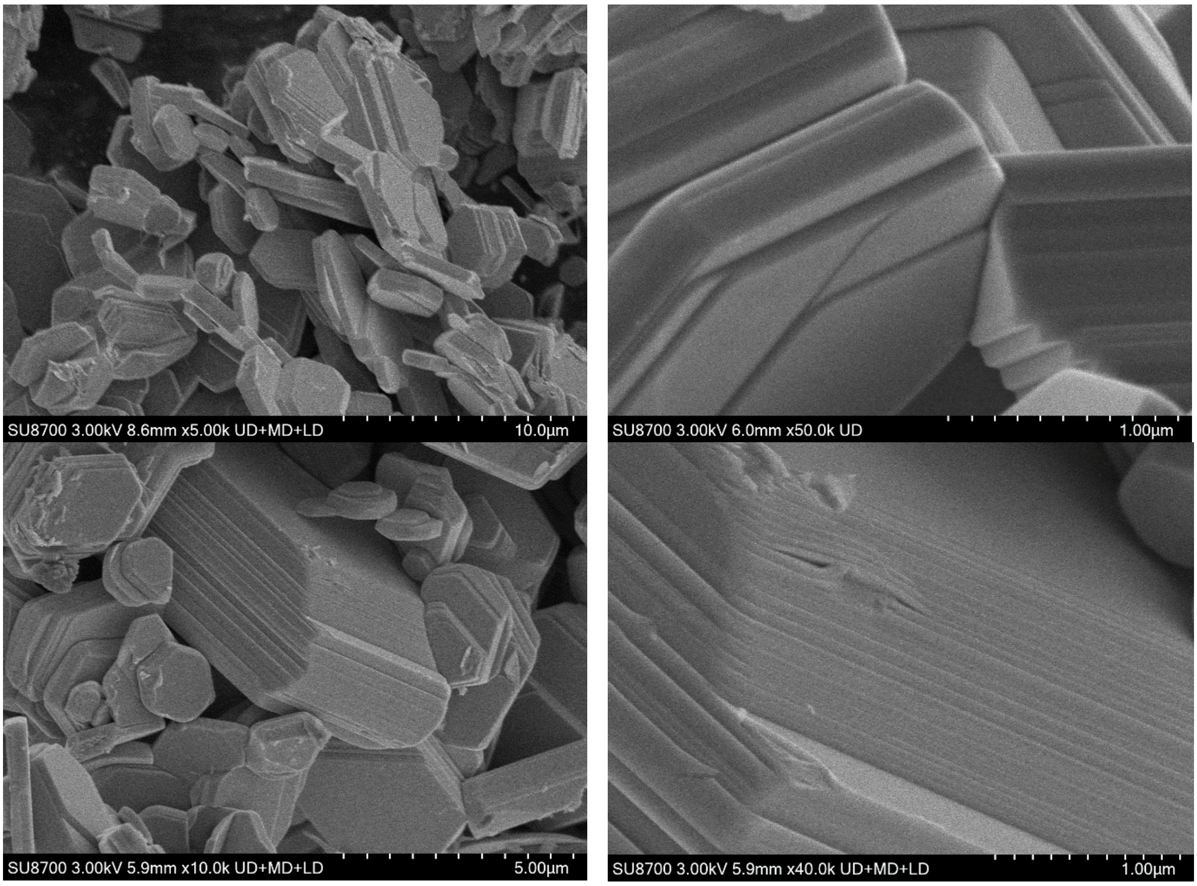

Figure S2: SEM images show hexagonal shaped crystallites with a layered structure in the pristine ZrS_2.

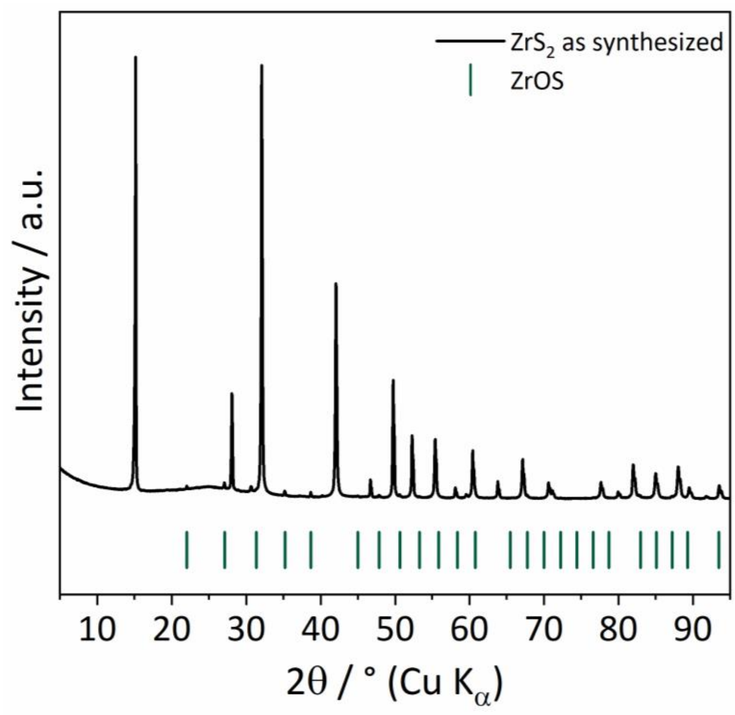

Figure S3: XRPD pattern of the synthesized and ball milled ZrS_2 sample (black) and the Bragg positions of the ZrOS secondary phase (green).

Detailed Diffraction pattern

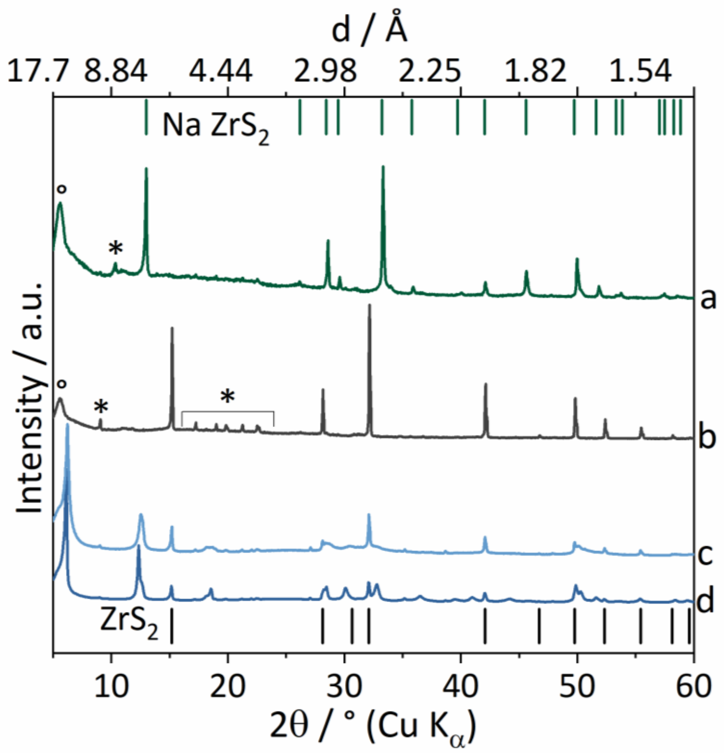

Figure S4: Long-term impact of the electrolyte NaOTf in diglyme on the as synthesized powders of $NaZrS_2$ (a) and ZrS_2 (b). Reflections caused by the Kapton foil (sample preparation) are marked with circles and reflections assigned to NaOTf are marked with asterisks. Further, we have measured one and the same sample of $Na_{0.1}ZrS_2$ after 3 years of relaxation time (c) and directly after disassembling from the electrochemical cell (d).

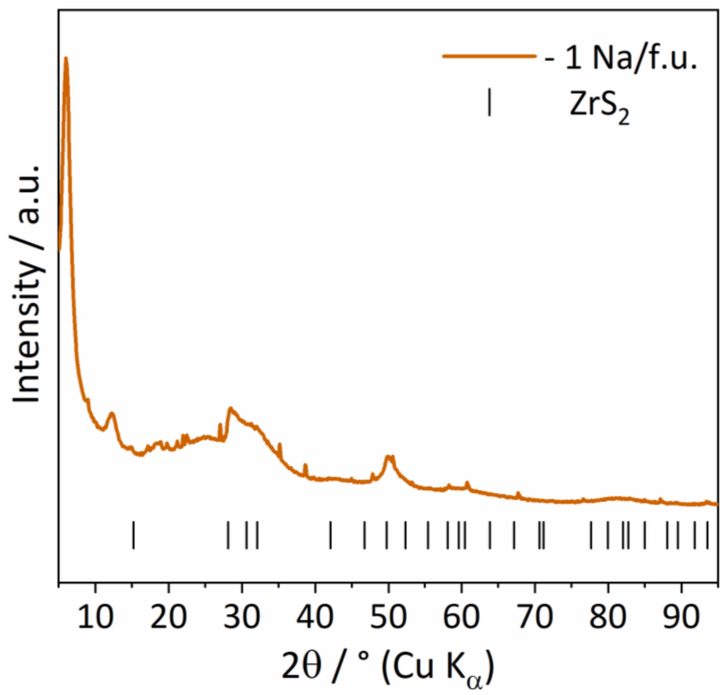

Figure S5: XRPD pattern after a full cycle of ZrS_2 vs. Na^+|Na (-1 Na^+/f.u.) using NaOTf in diglyme as electrolyte. A phase of low crystallinity is observed with a Warren type peak shape indicating significant turbostratic disorder of the layers.

Ex situ XRPD pattern of intercalation states

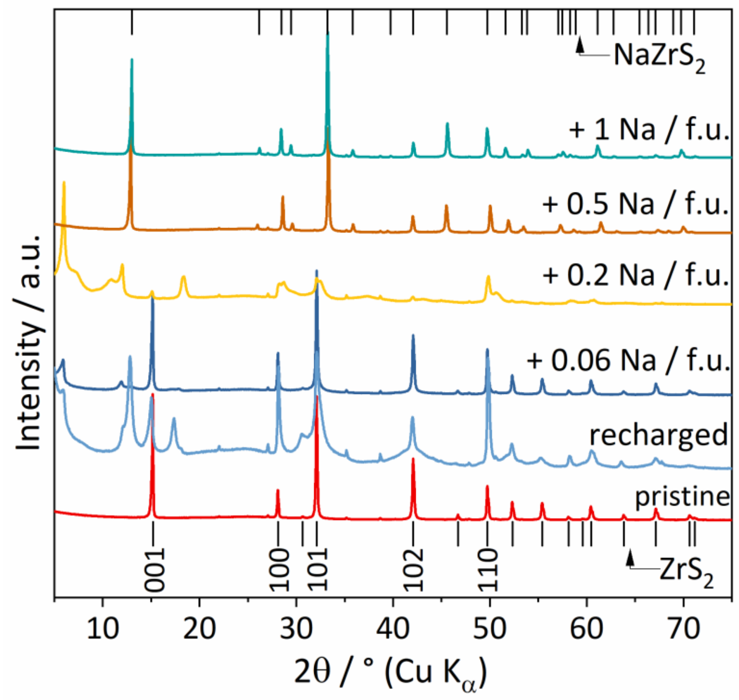

Figure S6: Ex situ XRPD pattern of ZrS_2 vs. Na|Na^+ using NaTFSI in EC:DEC (1:1) as electrolyte but washed with diglyme after (partly) de-/intercalation.

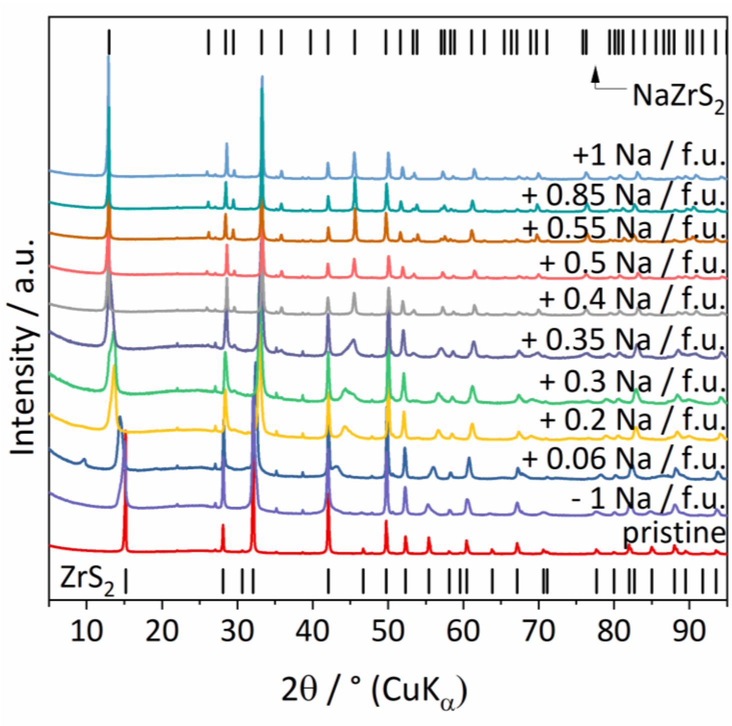

Figure S7: Complete range of ex situ XRPD pattern of different intercalation states from the stepwise uptake up to 1 Na/f.u. to the release of 1 Na/f.u. The cells consists of ZrS_2 vs. Na|Na^+ using NaTFSI in EC:DEC (1:1) as electrolyte.

EDX mapping

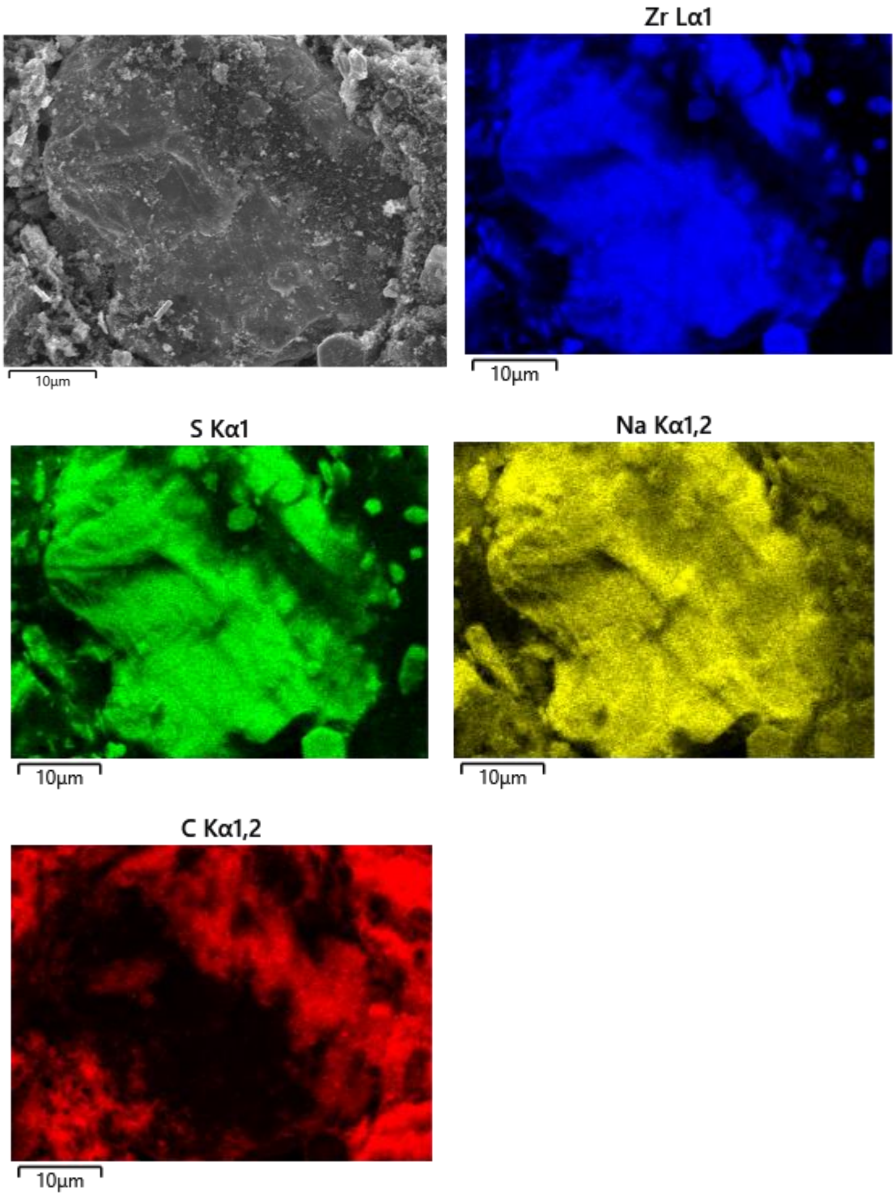

Figure S8: EDX mapping of $NaZrS_2$ show a uniform distribution of the elements Zr (blue), S (green), Na (yellow) and on the surface/aside from the $NaZrS_2$ crystals carbon (red).

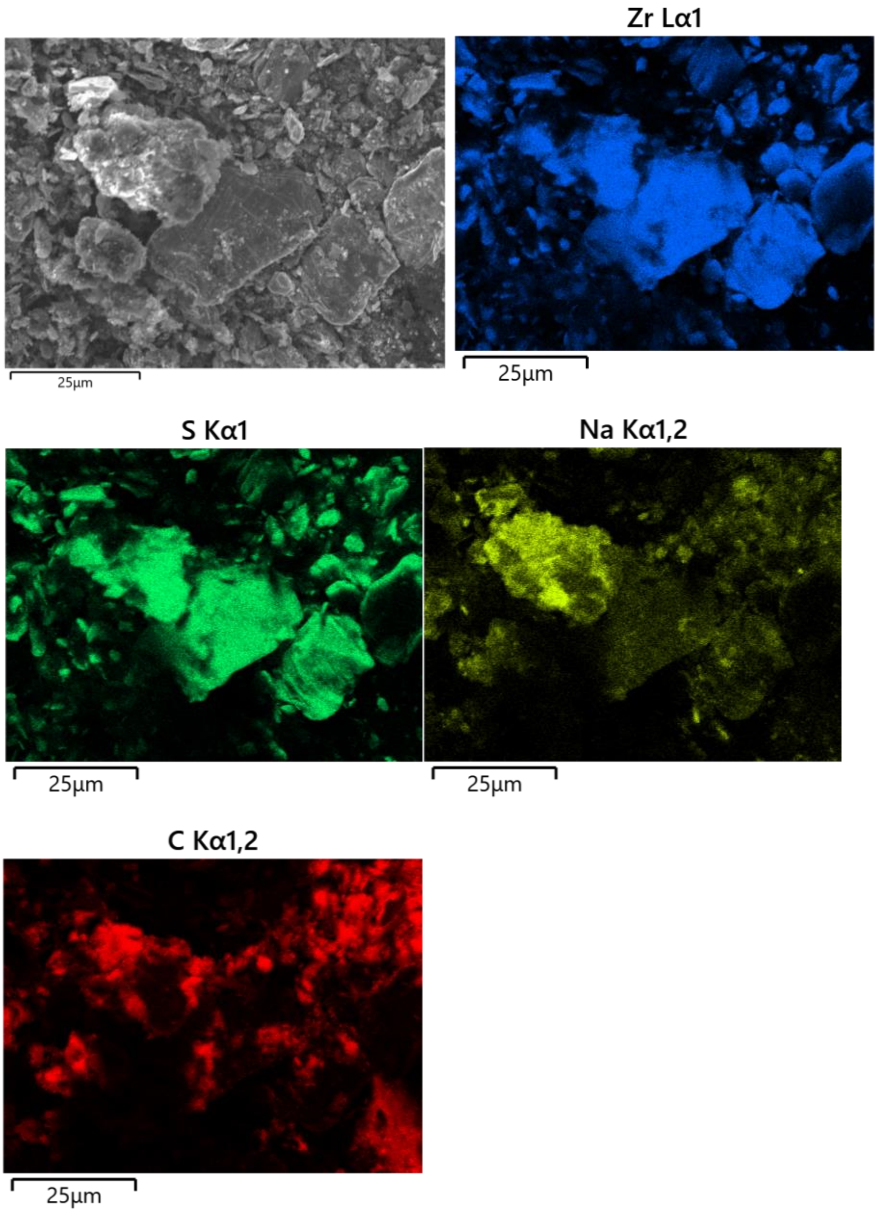

Figure S9: EDX mapping of deintercalated $(Na_x)ZrS_2$ show a uniform distribution of the elements Zr (blue), S (green) but also finely distributed sodium (yellow) on the sample. Carbon (red) embeds the sample.

Detailed analysis of the transition from 1T-ZrS_2 to 3R-Na_xZrS_2 via stacking faults

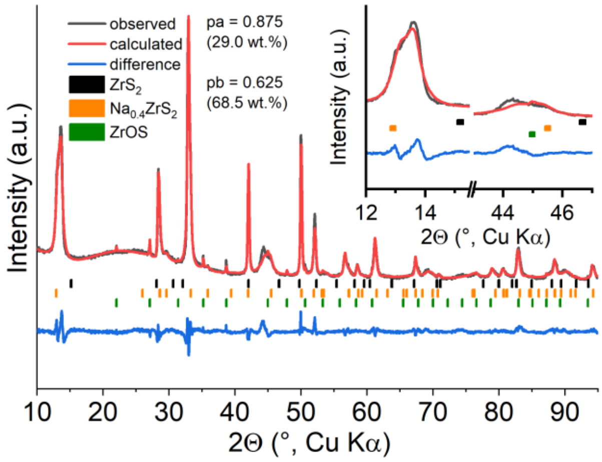

Figure S10: Difference plot (r_{wp} = 5.6 %) for the sample with 0.3 f.u. Na^+ intercalated (EC/DEC) using two separate stacking faulted phases with fault probabilities and weight fractions as indicated.

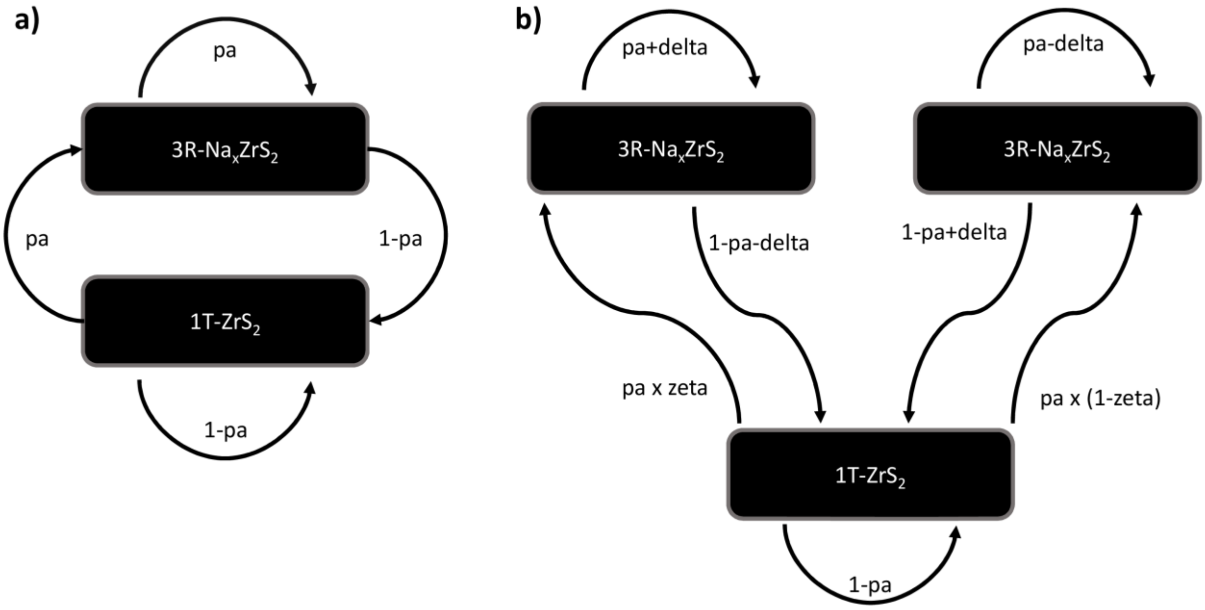

Figure S11: a) Scheme for fault probabilities with transitions from 1T-ZrS_2 to 3R-Na_xZrS_2. b) Extended model with transitions between 1T-ZrS_2 and two different domains of 3R-Na_xZrS_2 with distinct fault probabilities. Input files for TOPAS for both faulting scenarios are provided in the supporting files.

Table S2: Transition probability matrix with parameters as indicated in Figure S7 a).

From layer ↓ / To layer →	$1T-ZrS_2$	$3R-Na_xZrS_2$
$1T-ZrS_2$	1-pa	pa
$3R-Na_xZrS_2$	1-pa	pa

Table S3: Transition probability matrix with parameters as indicated in Figure S7 b).

From layer ↓ / To layer →	$1T-ZrS_2$	$3R-Na_xZrS_2$ (high)	$3R-Na_xZrS_2$ (low)
$1T-ZrS_2$	1-pa	pa x zeta	pa x (1-zeta)
$3R-Na_xZrS_2$ (high)	1-pa-delta	pa + delta	-
$3R-Na_xZrS_2$ (low)	1-pa+delta	-	pa-delta

Table S4: Transition probability matrix filled with the values obtained for the sample containing 0.2 f.u. of Na^+ (EC/DEC).

From layer ↓ / To layer →	$1T-ZrS_2$	$3R-Na_xZrS_2$ (high)	$3R-Na_xZrS_2$ (low)
$1T-ZrS_2$	0.33	0.1474	0.5226
$3R-Na_xZrS_2$ (high)	0.19	0.81	-
$3R-Na_xZrS_2$ (low)	0.47	-	0.53

Table S5: Transition probability matrix filled with the values obtained for the sample containing 0.3 f.u. of Na^+ (EC/DEC).

From layer ↓ / To layer →	$1T-ZrS_2$	$3R-Na_xZrS_2$ (high)	$3R-Na_xZrS_2$ (low)
$1T-ZrS_2$	0.27	0.0876	0.6424
$3R-Na_xZrS_2$ (high)	0.1	0.9	-
$3R-Na_xZrS_2$ (low)	0.44	-	0.56

Table S6: Transition probability matrix filled with the values obtained for the sample containing 0.35 f.u. of Na^+ (EC/DEC).

From layer ↓ / To layer →	1T-ZrS_2	3R-Na_xZrS_2 (high)	3R-Na_xZrS_2 (low)
1T-ZrS_2	0.18	0.1804	0.6396
3R-Na_xZrS_2 (high)	0.06	0.94	-
3R-Na_xZrS_2 (low)	0.3	-	0.7

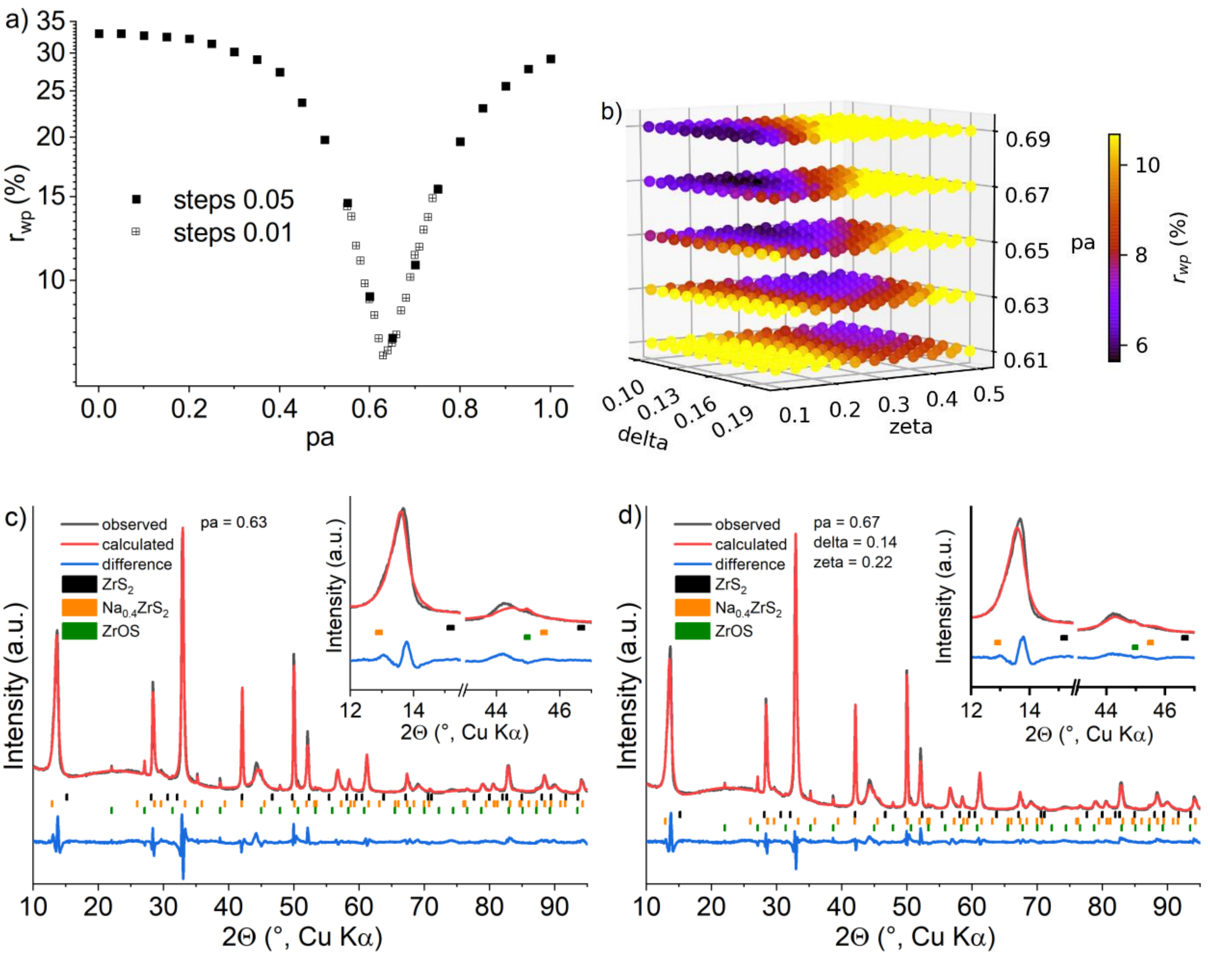

Figure S12: a) Linear optimisation of the fault probability of the sample with 0.2 f.u. of Na^+ intercalated. For the optimum pa the Rietveld refinement is shown (R_{wp} = 6.3 %) in c). The result for the three dimensional grid search on the extended structure model is shown in b), along with the final Rietveld plot in d) using the optimum values (R_{wp} = 5.2 %).

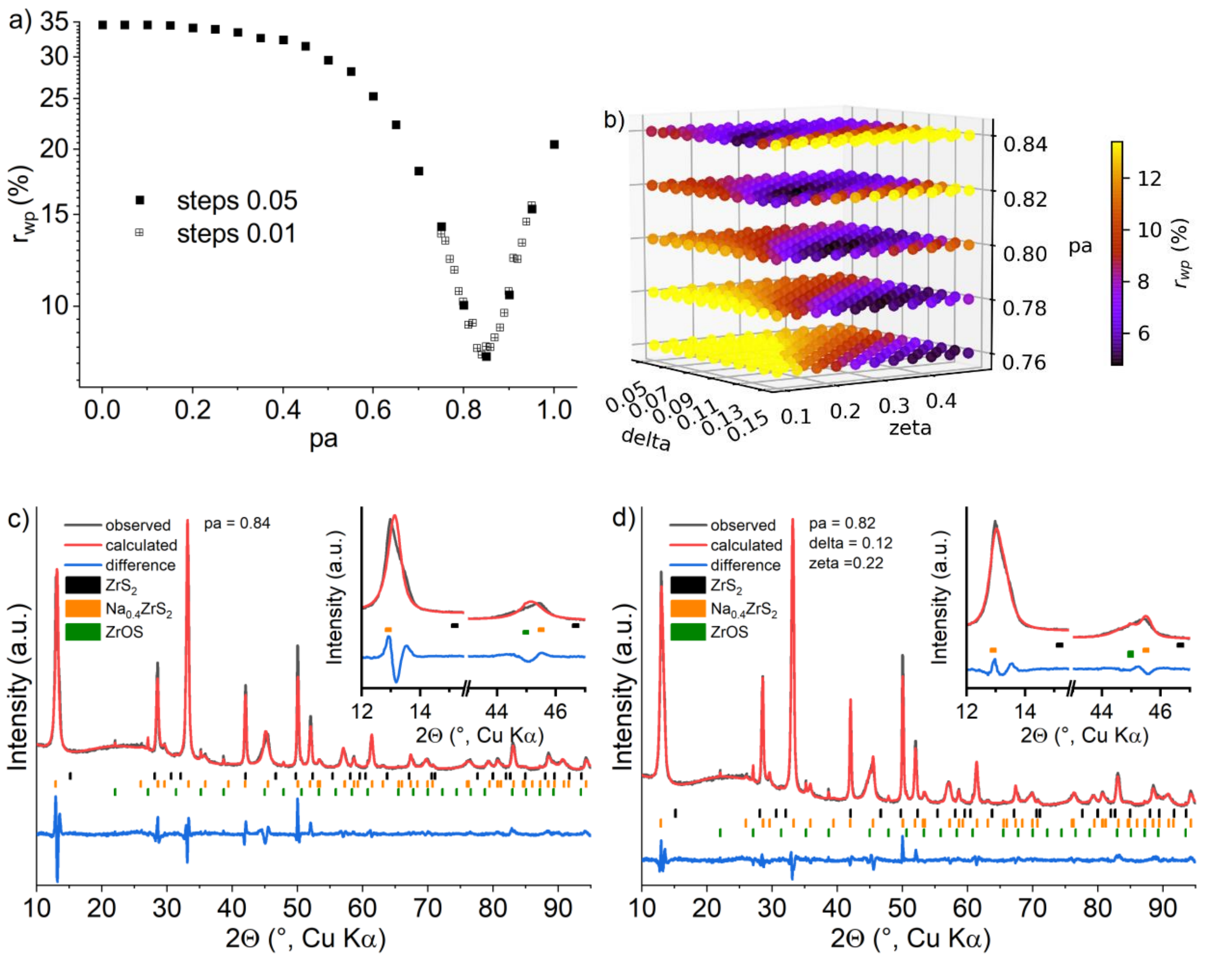

Figure S13: a) Linear optimisation of the fault probability of the sample with 0.35 f.u. of Na^+ intercalated. For the optimum pa the Rietveld refinement is shown (Rwp = 7.4 %) in c). The result for the three dimensional grid search on the extended structure model is shown in b), along with the final Rietveld plot in d) using the optimum values (R_{wp} = 4.5 %).

A general problem of these (linear and multi-dimensional) grid searches in TOPAS is the variation in R_{wp} when making multiples calls to the program with the same parameters for the stacking faults.[3] This is rooted in the stacking sequence being generated by a random number generator. The sequences generated have overall the same number of stacking faults each time, but the sequence of layers is different with each call. This can be mitigated by the *seed* keyword with a fixed number in the input file executed by TOPAS. Further, using a high number of stacks and a decent size of the stacks is recommended, although increasing the number here is computationally more expensive and therefore a trade off has to be made. Also reducing the number of refineable parameters improves to reproducibility in R_{wp}, in particular using a fixed background function (assuming it is well placed!) greatly helps.

In situ X-ray diffraction of a ZrS_2 vs. Na|Na^+ cell using diglyme as electrolyte solvent

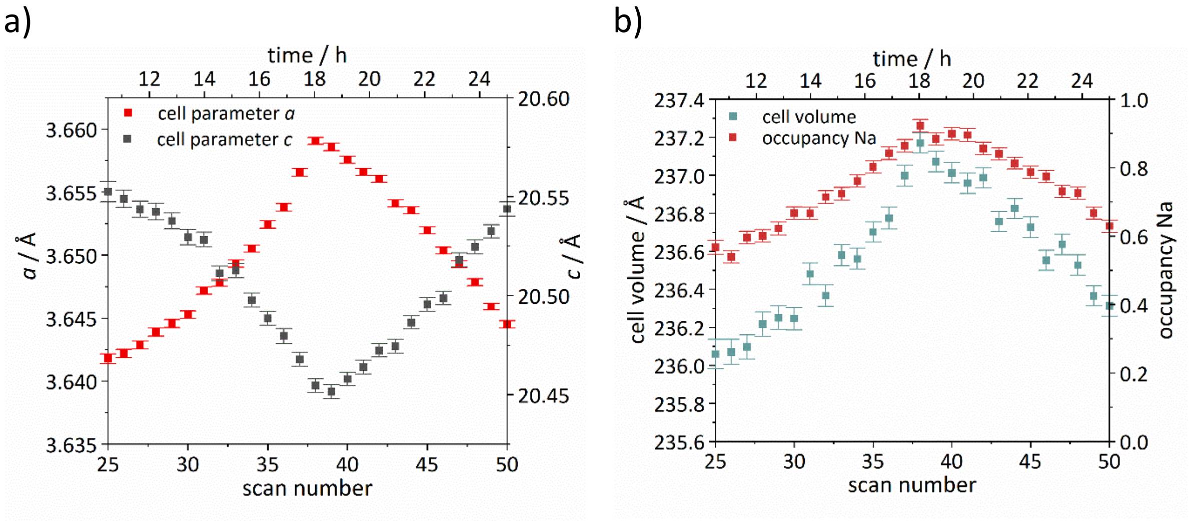

Figure S14: Sequential Rietveld refinement of a) cell parameter *a* and *c* and b) cell volume in correlation to intercalated sodium ions between 6 h and 17 h of cycling of ZrS_2 vs Na|Na^+ using NaTFSI in EC:DEC as electrolyte.

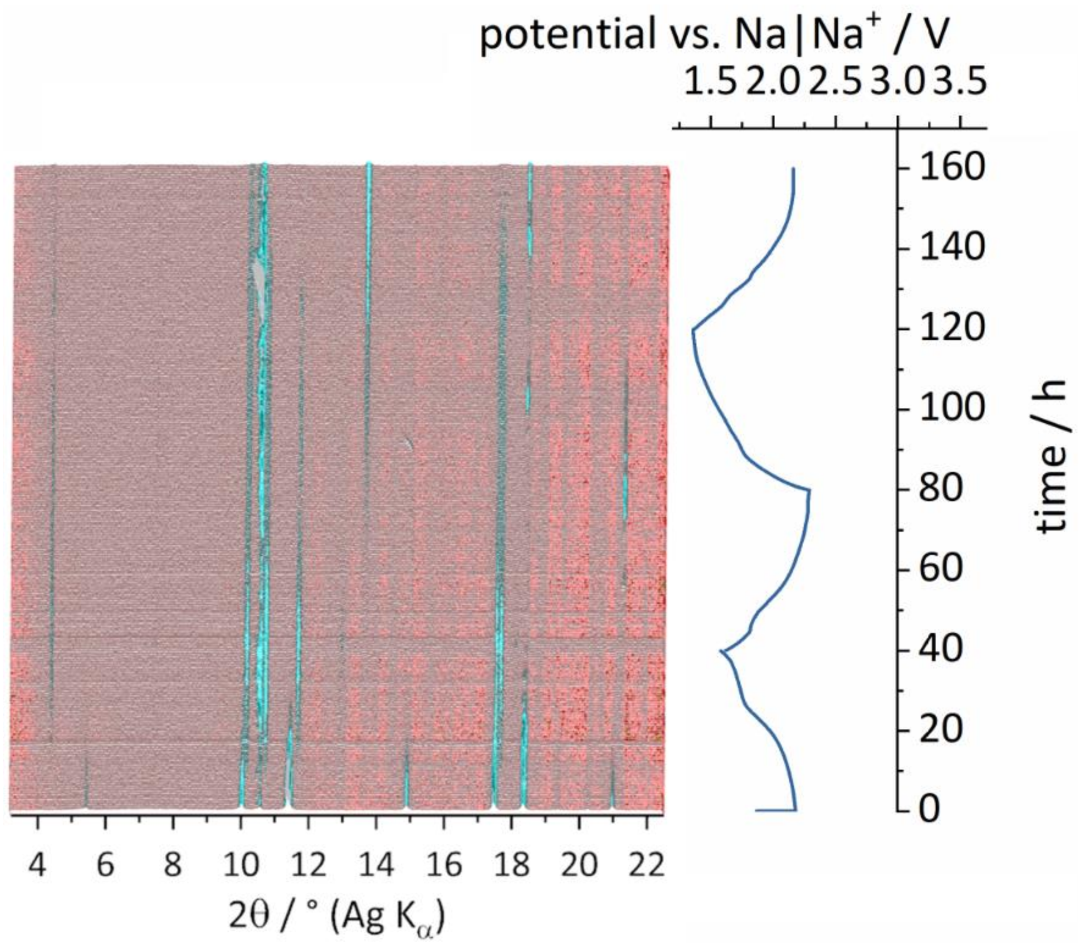

Figure S15: In situ XRPD of the first two full cycles of ZrS_2 vs. Na|Na^+ cells using NaOTf in diglyme as electrolyte (left) and the corresponding voltage profile of voltage vs. time (right).

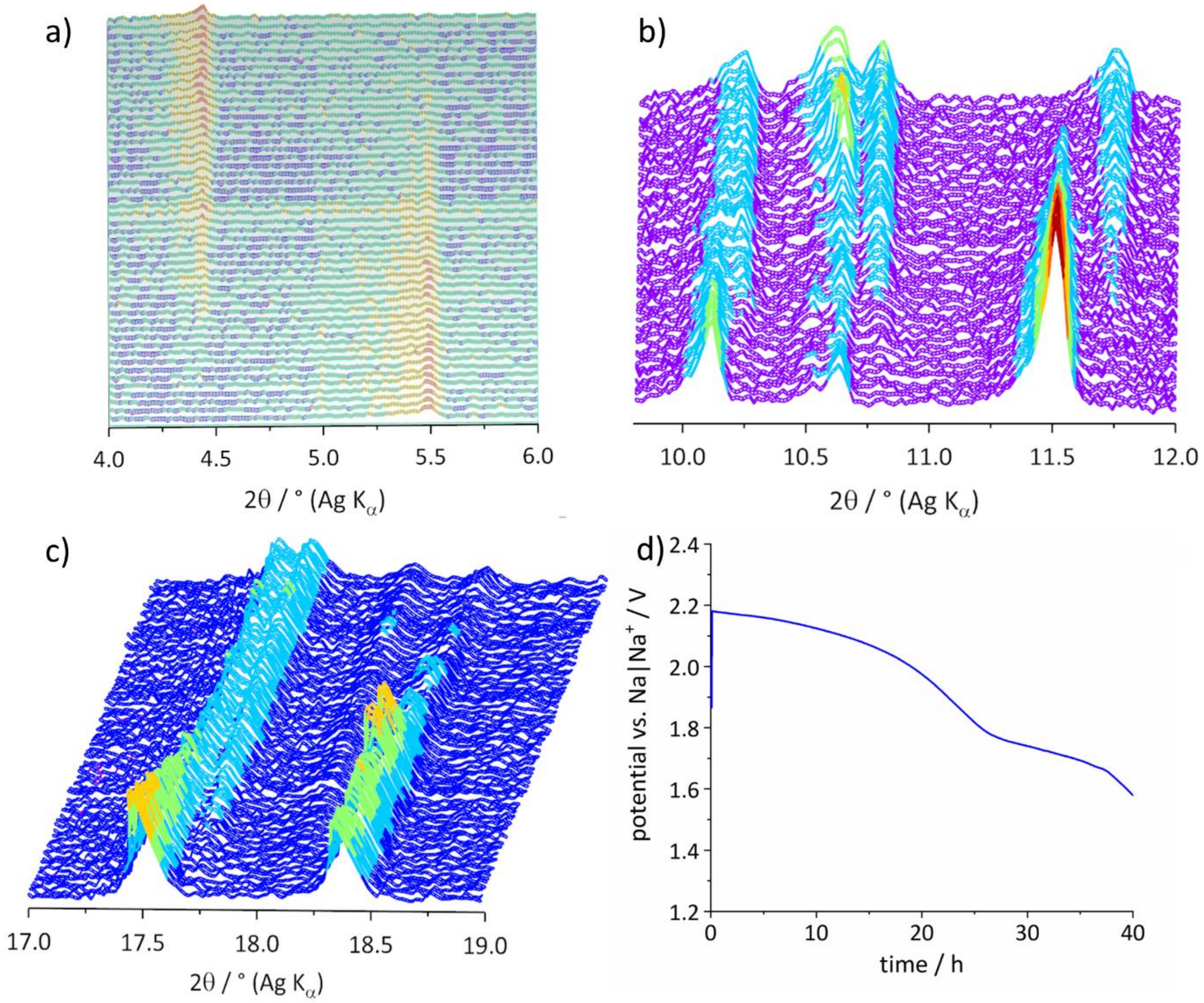

Figure S16: Excerpts of the most important diffraction angle ranges within the first discharge of a cell using a diglyme based electrolyte. The individual excerpts show reflection shifts of the 001 reflection (a), 100 and 101/011 reflection (b) and 110 and 111 reflection (c). Further, the voltage profile of the first discharge vs. the time is depicted (d).

Solid state ^{23}Na nuclear magnetic resonance spectroscopy

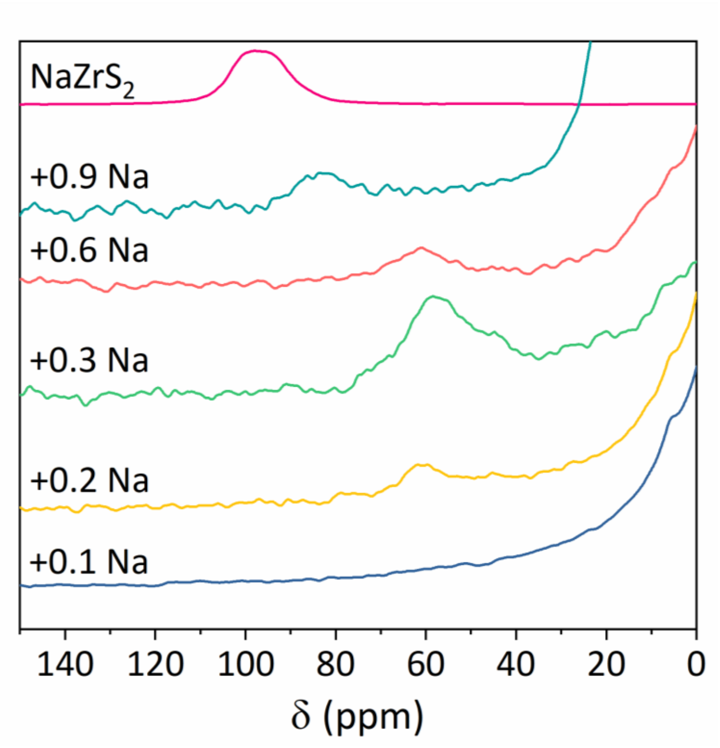

Figure S17: Detailed view of the ^{23}Na MAS NMR spectra of a chemical shift between 0 and 150 ppm of ZrS_2 for different intercalation states using an EC:DEC based electrolyte.

Electrochemical cycling of alternative electrolytes and long-time stability tests

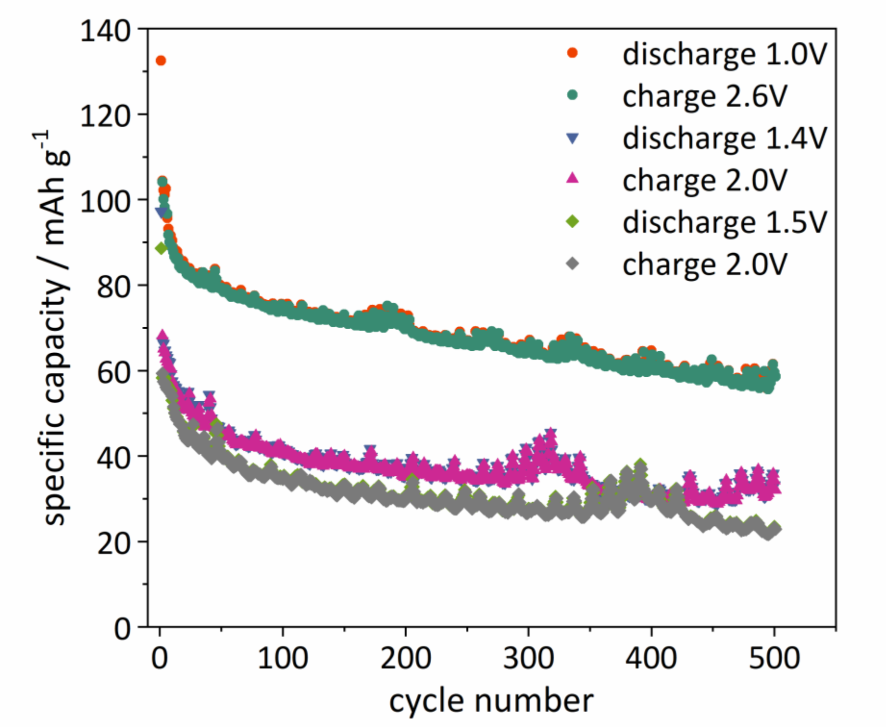

Figure S18: Cycle stability of EC:DEC based test cells cycled over 500 cycles at different voltage ranges. The discharge capacity is marked as red dots (1 V), blue triangels (1.4 V), light green diamonds (1.5 V) and the charge capacity is marked as green dots (2.6 V), pink triangles and grey diamonds (2.0 V, respectively).

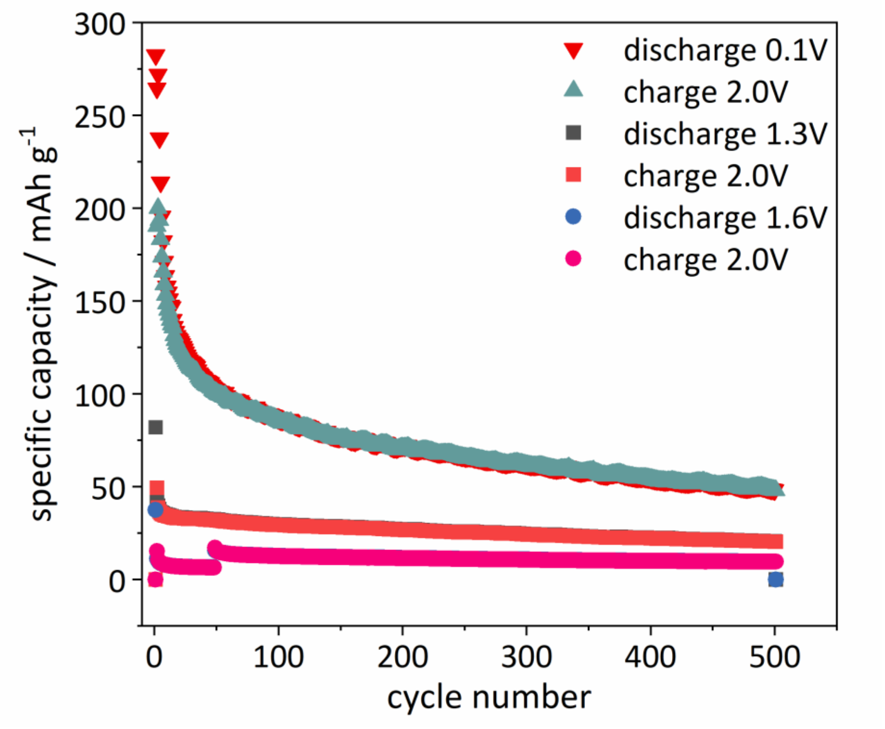

Figure S19: Cycle stability of diglyme based test cells cycled over 500 cycles at different voltage ranges. The discharge capacity is marked as red triangles (0.1 V), grey squares (1.3 V), blue dots (1.6 V) and the charge capacity is marked as green triangles, red squares and pink dots (2.0 V each).

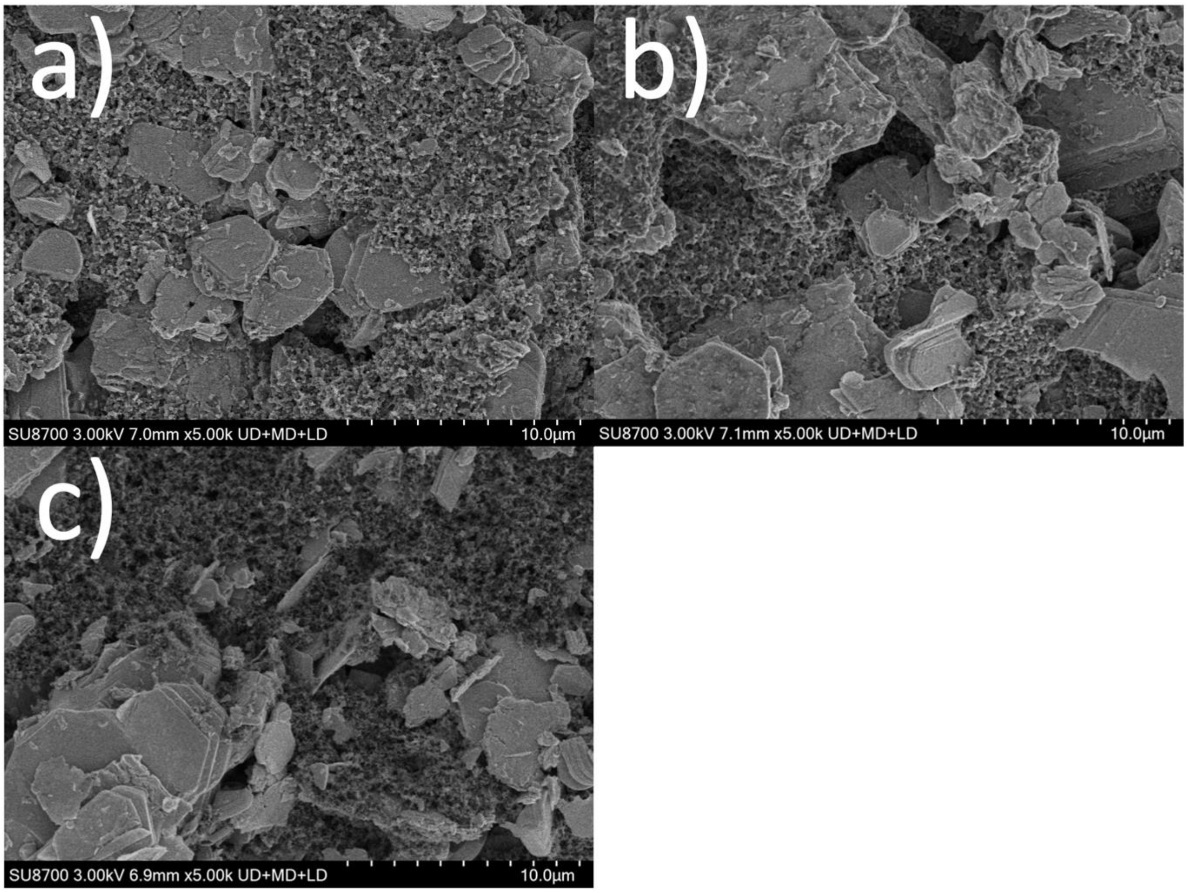

Figure S20: SEM images of electrodes before (a), after the 10th discharge (b) and after the 10th charge (c). No significant differences in the morphology or crystallinity were observed.

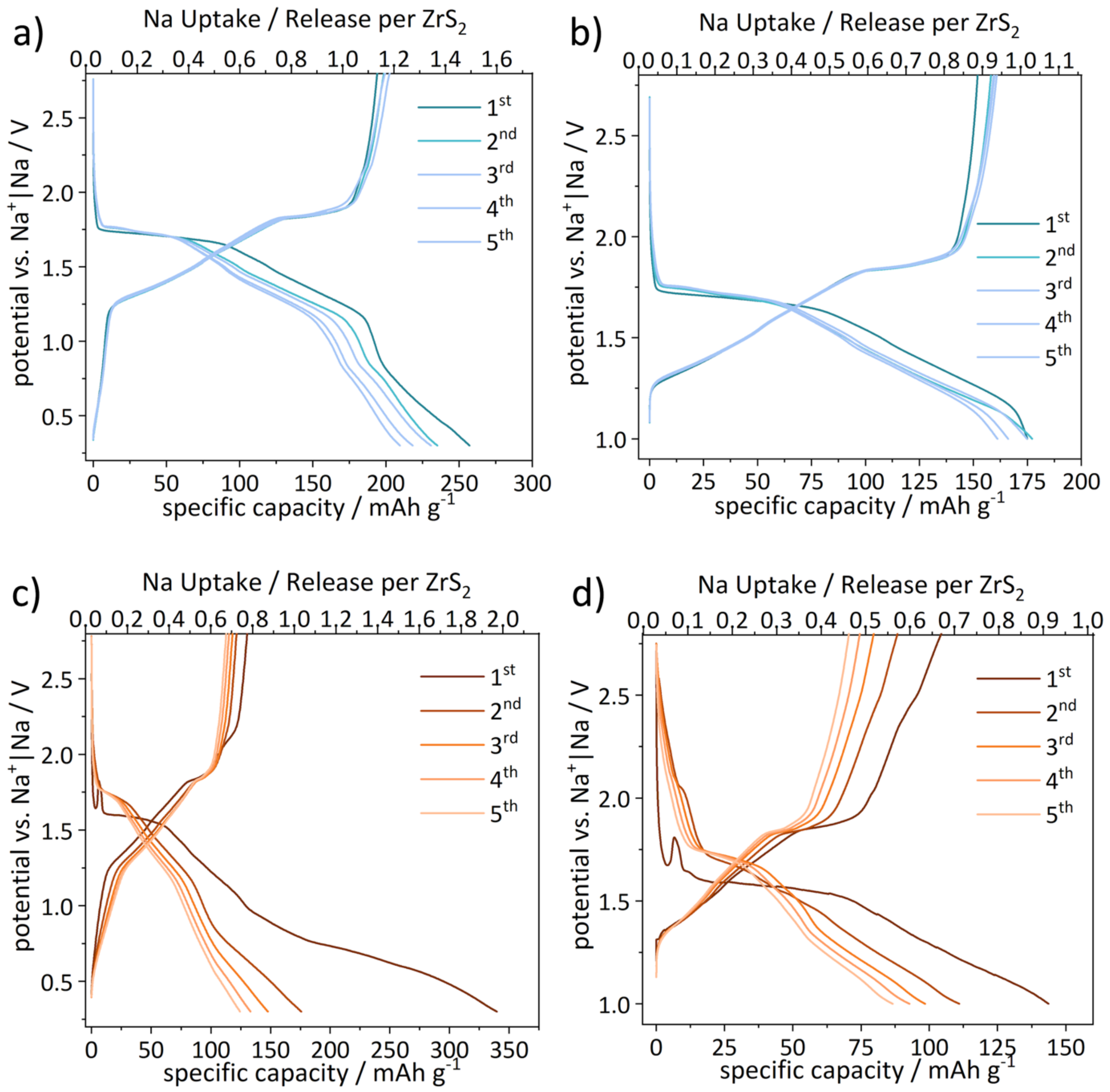

Figure S21: a and b) Voltage profiles of the first 5 cycles of test cells using a THF based electrolyte, c and d) a PC based electrolyte in a potential range of 0.3 - 2.8 V (left) and 1 - 2.8 V (right), respectively.
